# A new activity model for Mg–Al biotites determined through an integrated approach

**DOI:** 10.1007/s00410-019-1606-2

**Published:** 2019-08-23

**Authors:** Edgar Dachs, Artur Benisek

**Affiliations:** Fachbereich Chemie und Physik der Materialien, Abteilung Mineralogie, Universität Salzburg, Jakob-Haringerstrasse 2a, 5020 Salzburg, Austria

**Keywords:** Calorimetry, Heat capacity, Mg–Al biotite, Activity model, Thermodynamic mixing properties, Entropy, Phlogopite, Eastonite

## Abstract

A new activity model for Mg–Al biotites was formulated through an integrated approach combining various experimental results (calorimetry, line-broadening in infrared (IR) spectra, analysis of existing phase-equilibrium data) with density functional theory (DFT) calculations. The resulting model has a sound physical-experimental basis. It considers the three end-members phlogopite (Phl, KMg_3_[(OH)_2_AlSi_3_O_10_]), ordered eastonite (Eas, KMg_2_Al[(OH)_2_Al_2_Si_2_O_10_]), and disordered eastonite (dEas) and, thus, includes Mg–Al order–disorder. The DFT-derived disordering enthalpy, Δ*H*_dis_, associated with the disordering of Mg and Al on the M sites of Eas amounts to 34.5 ± 3 kJ/mol. Various biotite compositions along the Phl–Eas join were synthesised hydrothermally at 700 °C and 4 kbar. The most Al-rich biotite synthesized had the composition *X*_Eas_ = 0.77. The samples were characterised by X-ray diffraction (XRD), microprobe analysis and IR spectroscopy. The samples were studied further using relaxation calorimetry to measure their heat capacities (*C*_p_) at temperatures from 2 to 300 K and by differential scanning calorimetry between 282 and 760 K. The calorimetric (vibrational) entropy of Phl at 298.15 K, determined from the low-*T C*_p_ measurements on a pure synthetic sample, is *S*_cal_ = 319.4 ± 2.2 J/(mol K). The standard entropy, *S*^o^, for Phl is 330.9 ± 2.2 J/(mol K), which is obtained by adding a configurational entropy term, *S*_cfg_, of 11.53 J/(mol K) due to tetrahedral Al-Si disorder. This value is ~1% larger than those in different data bases, which rely on older calorimetrical data measured on a natural near-Phl mica. Re-analysing phase-equilibrium data on Phl + quartz (Qz) stability with this new *S*^o^, gives a standard enthalpy of formation of Phl, $\Delta H^{\text{o}}_{\text{f}} ,_{\text{Phl}}$ = − 6209.83 ± 1.10 kJ/mol, which is 7–8 kJ/mol less negative than published values. The superambient *C*_p_ of Phl is given by the polynomial [J/(mol K)] as follows: $C_{\text{p}} = 667.37\left( { \pm 7} \right) - 3914.50\left( { \pm 258} \right) \cdot T^{ - 0.5} - 1.52396\left( { \pm 0.15} \right) \times 10^{7} \cdot T^{ - 2} + \, 2.17269\left( { \pm 0.25} \right) \times 10^{9} \cdot T^{ - 3}$. Calorimetric entropies at 298.15 K vary linearly with composition along the Phl–Eas join, indicating ideal vibrational entropies of mixing in this binary. The linear extrapolation of these results to Eas composition gives *S*^o^ = 294.5 ± 3.0 J/(mol K) for this end-member. This value is in excellent agreement with its DFT-derived *S*^o^, but ~ 8% smaller than values as appearing in thermodynamic data bases. The DFT-computed superambient *C*_p_ of Eas is given by the polynomial [in J/(mol K)] as follows: $C_{\text{p}} = 656.91\left( { \pm 14} \right) - 3622.01\left( { \pm 503} \right) \cdot T^{ - 0.5} - 1.70983\left( { \pm 0.33} \right) \times 10^{7} \cdot T^{ - 2} + \, 2.31802\left( { \pm 0.59} \right) \times 10^{9} \cdot T^{ - 3}$. A maximum excess enthalpy of mixing, Δ*H*_*ex*_, of ~6 kJ/mol was derived for the Phl–Eas binary using line-broadening from IR spectra (wavenumber region 400–600 cm^−1^), which is in accordance with Δ*H*_ex_ determined from published solution-calorimetry data. The mixing behaviour can be described by a symmetric interaction parameter $W^{\text{H}}_{{{\text{Phl}},{\text{Eas}}}}$ = 25.4 kJ/mol. Applying this value to published phase-equilibrium data that were undertaken to experimentally determine the Al-saturation level of biotite in the assemblage (Mg–Al)-biotite-sillimanite-sanidine-Qz, gives a $\Delta H^{\text{o}}_{{{\text{f}},{\text{Eas}}}}$ = − 6358.5 ± 1.4 kJ/mol in good agreement with the independently DFT-derived value of ${\Delta H^{\rm o}_{\rm f,Eas}}^{\rm DFT}$ = − 6360.5 kJ/mol. Application examples demonstrate the effect of the new activity model and thermodynamic standard state data, among others, on the stability of Mg–Al biotite + Qz.

**Electronic supplementary material:**

The online version of this article (10.1007/s00410-019-1606-2) contains supplementary material, which is available to authorized users.

## Introduction

Biotite is a major rock-forming mineral that occurs in various igneous and particularly metamorphic rocks over a wide range of bulk compositions and metamorphic grades. Its thermodynamic properties are thus required in many petrological applications like phase-diagram calculations and geothermobarometry. One of the most prominent examples in this context is certainly the garnet-biotite geothermometer that has been widely used for several decades in metamorphic petrology and that experienced numerous calibrations and modifications over time. Most natural biotites are dominated by the two endmembers annite (Ann), KFe_3_[(OH)_2_AlSi_3_O_10_], and phlogopite (Phl), KMg_3_[(OH)_2_AlSi_3_O_10_], but, depending on *P* and *T* of formation and paragenetic relationships, the Tschermak substitution also plays an important role, leading to the Al-rich end-members eastonite (Eas), KMg_2_Al[(OH)_2_Al_2_Si_2_O_10_], and siderophyllite (Sid), KFe_2_Al[(OH)_2_Al_2_Si_2_O_10_]. Natural biotites incorporate further non-negligible amounts of Ti and Fe^3+^.

Calorimetric measurements on biotites are rare compared to the numerous phase-equilibrium and crystal-chemical investigations. A comprehensive list of the latter would be too long to be cited here, but see, e.g. Bailey (1984), Hewitt and Wones (1984) and Guidotti (1984) for a review of the earlier works, or Dachs and Benisek (2015) for the Fe-system. In this system, Hemingway and Robie (1990) measured the heat capacity of a natural aluminous ‘annite’ using low-temperature adiabatic calorimetry (low-TAC) and differential scanning calorimetry (DSC). The *C*_p_ of several synthetic members of the Ann-Sid join was measured by Benisek et al. (1999) over the temperature range 143–623 K using DSC. Dachs and Benisek (2015) applied relaxation calorimetry and DSC to provide *C*_p_ of a synthetic Ann in the *T*-range from 2 to 363 K and to extract its third law entropy. This Ann contained 10% Fe^3+^, which represents the closest possible synthetic composition to ‘ideal’ Ann.

In the Mg-system, Robie and Hemingway (1984) measured *C*_p_ on a natural near-phlogopite mica using low-TAC and DSC that deviated from the ideal Phl formula by the presence of FeO, TiO_2_ and F impurities. They extracted a calorimetric entropy, *S*_cal_, of 315.9 ± 1.0 J/(mol K) from their data. *S*^o^ values of Phl given in the thermodynamic data base of Holland and Powell (2011) or appearing in Berman et al. (2007) rely on this Robie and Hemingway value adding a configurational entropy contribution.

Circone and Navrotsky (1992) undertook a heat of solution study on synthetic members of the Phl–Eas join and found a considerable positive asymmetric deviation from ideality for the enthalpic mixing behaviour. Their most aluminous Mg–Al biotite synthesized had the composition *X*_Eas_ = 0.92. For representing their excess enthalpy of mixing (Δ*H*_ex_) data along the join, where Δ*H*_ex_ = Δ*H*_measured_ − ($\Delta H^{\text{o}}_{{{\text{f}},{\text{Phl}}}}$·(1 − *X*_Eas_) + $\Delta H^{\text{o}}_{{{\text{f}},{\text{Eas}}}}$·*X*_Eas_), they derived the interaction parameters *W*_PhlEas_ = 66.3 ± 17.3 kJ/mol and *W*_EasPhl_ = 0.4 ± 43.2 kJ/mol. As noted by Circone and Navrotsky (1992), a regular mixing model, on the other hand, would give *W*_H_ = 22.8 ± 18.7 kJ/mol, but does not account for the asymmetry in their data. By treating the most aluminous biotite data point of Circone and Navrotsky (1992) as outlier, Holland and Powell (1998) argued for a symmetric *W*_Phl,Eas_ = 10 ± 4 kJ/mol for the Phl–Eas join. This value was used in the subsequent K_2_O–FeO–MgO–Al_2_O_3_–SiO_2_–H_2_O (KFMASH) biotite activity models of Powell and Holland (1999) and Holland and Powell (2006), as well as in the more comprehensive models of White et al. (2000, 2007, 2014) and Tajčmanová et al. (2009). In these models it is assumed that octahedral Al preferentially partitions onto the M1 octahedral site in biotite (*trans*-coordinated by hydroxyl groups) and not onto the two equivalent M2 octahedral sites (*cis*-coordinated by hydroxyl groups, Mercier et al. 2005, 2006).

By omitting the same most aluminous sample of Circone and Navrotsky (1992) and noting that the other data are linear with composition up to *X*_Eas_ = 0.8, Berman et al. (2007) argued for ideal mixing along the Phl–Eas join. The Al solubility in biotite in the water-saturated assemblage biotite + sillimanite + sanidine + quartz has been determined in this relatively recent study from reversed phase-equilibrium experiments.

In this study we focus on the Phl–Eas join. We apply an integrated approach combining results from calorimetry, from density functional theory (DFT) calculations, from line-broadening in IR spectra and from evaluation of phase-equilibrium data to provide the following:

1. a revised *S*^o^ value for Phl, based on relaxation calorimetric measurements on synthetic pure Phl, and a revised enthalpy of formation value ($\Delta H^{\text{o}}_{\text{f}}$) for this end-member, based on its revised *S*^o^ and an evaluation of phase-equilibrium data on Phl + Qz stability (Bohlen et al. 1983; Aranovich and Newton 1998; Berman et al. 2007),
2. reliable thermodynamic data for the Eas end-member,
3. a new activity model for Mg–Al biotite based on Mg–Al ordering that is consistent with all information stemming from the physical/experimental branches calorimetry, IR spectroscopy, DFT calculations and phase-equilibrium experiments.

In a forthcoming paper we extend this new Mg–Al biotite activity model to a more comprehensive one that can then be used in petrological calculations involving biotite in general.

## Methods

### Sample synthesis and characterisation techniques

Samples along the phlogopite (Phl)–eastonite (Eas) binary were synthesized from gels in a conventional cold-seal hydrothermal apparatus at a temperature of 700 °C, pressure of 4 kbar and run durations of 3 weeks. Details of the hydrothermal apparatus, the applied gel method and chemicals used in the gel preparation can be found in Dachs (1994) and will not be repeated here. The samples calorimetrically studied will be subsequently referred to as, e.g. Phl50Eas50 for K(Mg_2.5_Al_0.5_)[(OH)_2_Al_1.5_Si_2.5_O_10_], or Phl100 for KMg_3_[(OH)_2_AlSi_3_O_10_]. The nominal compositions synthesized were Phl100, Phl80Eas20, Phl60Eas40, Phl50Eas50, Phl40Eas60, Phl20Eas80 and Eas100.

Synthesis products were examined optically and by XRD using a Bruker D8 advance to check phase purity. Their chemical composition was determined using an energy dispersive electron microscope (Zeiss Ultraplus 55 equipped with an Oxford Instrument 50 mm^2^ SDD EDX detector) for which the powder samples were prepared by pressing pellets.

Lattice constants were calculated from XRD patterns collected between 5° and 110° 2*θ* (Cu–K_α_ radiation) using the software UnitCell (Holland and Redfern 1997). The lattice constants determined in this way were checked by a Rietveld refinement (Fullprof, Rodriguez-Carvajal 2001) for the sample Phl60Eas40.

IR spectra were recorded on a Bruker IFS66v/S spectrometer in the wave number region 399–7500 cm^−1^ in order to investigate the line broadening as a result of forming the Phl–Eas solid solution.

### Calorimetric methods

Low-temperature heat capacities were measured using a commercially designed relaxation calorimeter (the heat capacity option of the Quantum Design^®^ Physical properties measurement system—PPMS) at Salzburg University. The data were collected in triplicate at 60 different temperatures between 2 and 300 K, using a logarithmic spacing so that the data density increased as the temperature decreased. The samples consisted of 11.1–13.6 mg crystallites wrapped in thin Al-foil and compressed to a ~0.5 mm-thick pellet that was then attached to the sample platform of the calorimeter with Apiezon N-grease, in order to facilitate the required thermal contact. The so-called sample coupling is a measure of the quality of the thermal contact between sample and sample platform (see, e.g. Dachs and Bertoldi 2005). It is defined as the ratio 100 *K*_g_/(*K*_g_ + *K*_w_), where *K*_g_ is the thermal conductance between the sample and the sample platform and *K*_w_ is the thermal conductance of the wires that attach the sample platform to the puck frame of the calorimeter. The closer this quantity is to 100%, the better the thermal conductance between sample and sample platform and the more reliable the heat capacity determination. Further details on the calorimetric technique and measuring procedures have already been described several times and will not be repeated here (e.g., Lashley et al. 2003; Dachs and Bertoldi 2005; Kennedy et al. 2007; Dachs and Benisek 2011).

Heat capacities around and above ambient *T* were collected using a Perkin Elmer Diamond DSC^®^. Further details on DSC measurement and calibration procedures have been previously published (e.g., Dachs and Benisek 2011; Benisek et al. 2012). All calorimetric data are given in the Supplementary Table S1.

### Evaluation of the calorimetric data

The calorimetric (vibrational) molar entropy (*S*_cal_) of each compound at 298.15 K was calculated by solving the following integral:

1 $$S_{{\text{cal}}} = S^{{T = 298.15\,{\text{K}}}} - S^{{T = 0\,{\text{K}}}} = \mathop{\smallint}\limits_{0}^{298.15} \frac{{C_{{\text{p}}}}}{T}{\text{d}}T,$$

where *S*_cal_ corresponds to the standard (third-law) entropy, *S*^o^, in the case of an ordered end-member (assuming *S*^*T*=0K^ = 0). The DSC *C*_*p*_ data were combined with the PPMS data around ambient temperature and fitted to a polynomial of the following form (Berman and Brown 1985):

2 $$C_{\text{p}} = k_{0} + \, k_{1} \cdot T^{ - 0.5} + \, k_{2} \cdot T^{ - 2} + k_{3} \cdot T^{ - 3} .$$

The entropy increment 0 K–2 K, not covered by measured C_p_ data, is assumed to be insignificant, because absolute *C*_p_ values are so small so that this increment affects *S*_cal_ only at the 2nd decimal place. Errors in *S*_cal_ were estimated according to Dachs and Benisek (2011).

### Computational methods

Quantum-mechanical calculations were based on the DFT plane wave pseudopotential approach implemented in the CASTEP code (Clark et al. 2005) included in the Materials Studio software from Accelrys^®^. The calculations used the local density approximation for the exchange-correlation functional (Ceperley and Alder 1980). To describe the core-valence interactions, norm-conserving pseudopotentials were used with the 1 s^1^, 2s^2^2p^4^, 2s^2^2p^6^3 s^2^, 3s^2^3p^1^, 3s^2^3p^2^ and 3s^2^3p^6^4 s^1^ electrons explicitly treated as valence electrons for H, O, Mg, Al, Si and K, respectively.The k-point sampling used a Monkhorst-Pack grid (Monkhorst and Pack 1976) with a spacing of 0.02 Å^−1^ for the energy calculations. Convergence was tested by performing calculations using a denser k-point grid. The structural relaxation was calculated applying the BFGS algorithm, where the maximum force on the atom was within 0.01 eV/Å. The lattice dynamical calculations were performed for the relaxed structures within the linear response approximation implemented in CASTEP using the interpolation approach and a wider k-point grid (spacing of 0.05 Å^−1^).The enthalpy of mixing was simulated by the single defect method (Sluiter and Kawazoe 2002), which investigates supercells with almost endmember composition having only a single substitutional defect. The energy calculations of the endmembers and such supercells give directly the knowledge of the interaction parameters, because the results can easily be transformed into the slopes of the heat of mixing function (Li et al. 2014).

## Results

### Sample characterization and biotite end-members

Both X-ray and optical microscope results indicate that the synthesis experiments were successful yielding the 1 M polytype form of biotite as the only phase in all syntheses with *X*_Eas_ ≤ 0.6 to which all Bragg peaks could be indexed. In syntheses with *X*_Eas_ > 0.6, corundum was detected in the XRD patterns as an impurity phase with the largest amount of perhaps around 10% in the Al-richest synthesis of nominal Eas100 composition.

A SEM image of Phl100 is shown as an example in Supplementary Fig. S1. The crystals appear as densely packed, several hundred μm sized agglomerates of finely crystallised, often pseudo-hexagonal platelets with diameters not exceeding 10 μm and submicrometer to maximal 1 μm thicknesses. Other phases could not be detected in this sample.

Formula units of all synthetic Phl–Eas micas are given in Table 1. They are the average of 20–30 microprobe analyses measured for each sample. Their scatter is larger than would be obtained with coarse-grained crystals due to the presence of void space between individual platelets, the large number of grain boundaries, uneven surfaces, etc. in the agglomerates. This leads to a relative error of 1–2% in formula units (Table 1), with a significantly larger error of around 4% for Eas100.

**Table 1:** Formula units, mole fractions of biotite end-members, molar weights, calorimetric (vibrational) entropies, *S*_cal_, at 298.15 (determined from PPMS measurements), Δcorr values and resulting Δ*H*_mix_ of synthetic members of the Phl–Eas join

	Phl100	Phl80Eas20	Phl60Eas40	Phl50Eas50	Phl40Eas60	Phl20Eas80	Eas100
Si	3.01 (3)	2.80 (2)	2.62 (2)	2.56 (2)	2.45 (3)	2.32 (3)	2.23 (8)
Al	1.02 (3)	1.47 (3)	1.87 (3)	1.91 (3)	2.16 (2)	2.39 (3)	2.54 (10)
Mg	2.99 (6)	2.75 (6)	2.47 (3)	2.48 (3)	2.39 (4)	2.30 (3)	2.19 (2)
K	0.97 (5)	0.92 (5)	0.95 (2)	0.95 (2)	0.99 (1)	0.93 (3)	1.00 (4)
Al (tet)	0.99 (3)	1.20 (2)	1.38 (2)	1.44 (2)	1.55 (3)	1.68 (3)	1.77 (8)
Al (oct)	0.03 (3)	0.27 (4)	0.49 (3)	0.47 (4)	0.62 (4)	0.71 (5)	0.77 (13)
Sum (oct)	3.02 (6)	3.02 (7)	2.96 (5)	2.95 (5)	3.01 (5)	3.01 (5)	2.96 (6)
*X*_Phl_	1.00 (1)	0.77 (2)	0.57 (3)	0.54 (2)	0.42 (2)	0.31 (2)	0.22 (5)
*X*_Eas_	0.00 (2)	0.21 (2)	0.39 (2)	0.43 (2)	0.56 (3)	0.69 (3)	0.76 (8)
*X*_Ms_	0.00 (2)	0.02 (2)	0.05 (2)	0.03 (2)	0.02 (2)	0.01 (3)	0.01 (5)
Sum (*X*)	1.00 (3)	1.00 (4)	1.00 (3)	1.00 (4)	1.00 (4)	1.00 (5)	1.00 (6)
*X*_Eas_/(*X*_Eas_+*X*_Phl_)	0.00 (2)	0.22 (2)	0.41 (1)	0.44 (2)	0.57 (2)	0.69 (2)	0.77 (5)
Molar weight	418.4 (8)	419.6 (15)	417.3 (7)	416.3 (19)	418.9 (28)	419.2 (12)	416.8 (31)
*S*_cal_ [J/(mol K)]	319.4 (22)	314.6 (22)	308.4 (22)	306.9 (22)	304.8 (21)	303.4 (21)	299.2 (21)
Δcorr_mid_^a^	34.31	43.46	51.59		62.79		48.78
Δ*H*_mix_^b^ (kJ/mol)		3.02	5.15		8.22		3.78
Δcorr_high_^a^	57.08	58.22	52.13		52.29		39.36
Δ*H*_mix_^b^ (kJ/mol)		1.88	1.83		3.16		1.69

Formula units were calculated from wt% of oxides, obtained by electron microprobe analyses, using an oxygen basis of 11. End-members are phlogopite (Phl, KMg_3_[(OH)_2_AlSi_3_O_10_]), eastonite (Eas, KMg_2_Al[(OH)_2_Al_2_Si_2_O_10_]) and muscovite (Ms, KAl_2_[(OH)_2_AlSi_3_O_10_]). Numbers in parenthesis represent one standard deviation of the mean (the number of analyses for each sample was in the range 20–30)

^a^Δcorr values obtained from line broadening in IR spectra (Fig. S1) in the wave number regions 400–600 cm^−1^ (Δcorr_mid_) and 790 – 1330 cm^−1^

(Δcorr_high_)

^b^ΔH_mix_ computed from Δcorr values based on the correlation given in Etzel and Benisek (2008)

The compositional data indicate that octahedral sums are 3.0 or close to 3.0 within error and thus that no or only small amounts of octahedral vacancies are present in the synthetic Mg–Al biotites. Formula units of K range between 0.93 and 1.0. The values < 1.0 are thought to rather represent an analytical artefact than the true presence of interlayer-vacancies due to the reasons mentioned above (voids, etc. in the fine-grained mica aggregates). We thus assume that interlayer sites are completely filled with K. Another mineral-chemical feature to be mentioned is the presence of excess octahedral Al in the amount of 0.03–0.11 per formula unit. This amount of Al^VI^ is thus not balanced by the Tschermak substitution.

Three end-members are required to represent the mineral-chemical analyses (Table 1). These are phlogopite (Phl, KMg_3_[(OH)_2_AlSi_3_O_10_]), eastonite (Eas, KMg_2_Al[(OH)_2_Al_2_Si_2_O_10_]) and a third component that accounts for the non-negligible amount of excess-Al^VI^. For explaining excess-Al^VI^ in natural biotites from pelitic rocks, possible substitutions discussed in the literature are (e.g., Guidotti 1984; Dymek 1983; Tracy 1978; Fletcher and Greenwood 1979; Konings et al. 1988):

3 $$3\left( {{\text{Mg}}^{ 2+ } } \right)^{\text{VI}} = 2\left( {{\text{Al}}^{ 3+ } } \right)^{\text{VI}} + {\text{vacancy}}^{\text{VI}} ,$$

4 $$\left( {{\text{Mg}}^{{2 + }} } \right)^{{{\text{VI}}}} + \left( {{\text{K}}^{ + } } \right)^{{{\text{XII}}}} = \left( {{\text{Al}}^{{3 + }} } \right)^{{{\text{VI}}}} \;{\text{ + vacancy}}^{{{\text{XII}}}}.$$

If interlayer vacancies were truly present in our synthetic biotites, substitution (4), which is a linear combination of a classical Tschermak and a K-edenite exchange, might be relevant to explain excess-Al^VI^, but for the reasons discussed above and from our experience with microprobe analyses on synthetic biotites, we think this is unlikely. We thus follow most workers in assuming that excess-Al^VI^ is incorporated via a dioctahedral muscovite component (Ms, KAl_2_[(OH)_2_AlSi_3_O_10_]) in some of our synthetic biotites. These are thus strictly not binary Phl–Eas solid solution members, but some have an additional Ms component in the order of 1–5 mol%. The calculated end-member mole fractions have an uncertainty of ± 2–3 mol%, as computed by error propagation from the uncertainties of formula units. They show that Phl100, Phl80Eas20, Phl60Eas40 and Phl40Eas60 are on-composition within error (Table 1). The two most Al-rich samples Phl20Eas80 and Eas100, on the other hand, have considerably less Eas component than expected from their nominal composition. The Phl20Eas80 gel produced a Mg–Al biotite with *X*_Eas_ = 0.69 and the Eas100 gel a biotite with *X*_Eas_ = 0.77, which is the most Al-rich composition of our study. Hewitt and Wones (1975) estimated that the maximum Al^VI^ content corresponds to *X*_Eas_ = 0.62 in their synthetic Mg–Al biotites, whereas Circone et al. (1991) found a corresponding limit of *X*_Eas_ = 0.92. As mentioned above, the XRD patterns of these two samples showed that these are not pure phases, but in fact a multi-phase assemblage of Mg–Al biotite, corundum and probably sanidine (though not detected), with the largest amount of corundum in Eas100. The biotite formula units in this sample have consequently the largest errors, because part of the microprobe analyses represent mixed analyses resulting in a larger data scatter.

### End-member thermodynamic properties of Phl and Eas

#### Low-temperature heat capacity and standard entropy

##### Phlogopite

**Fig. 1:**
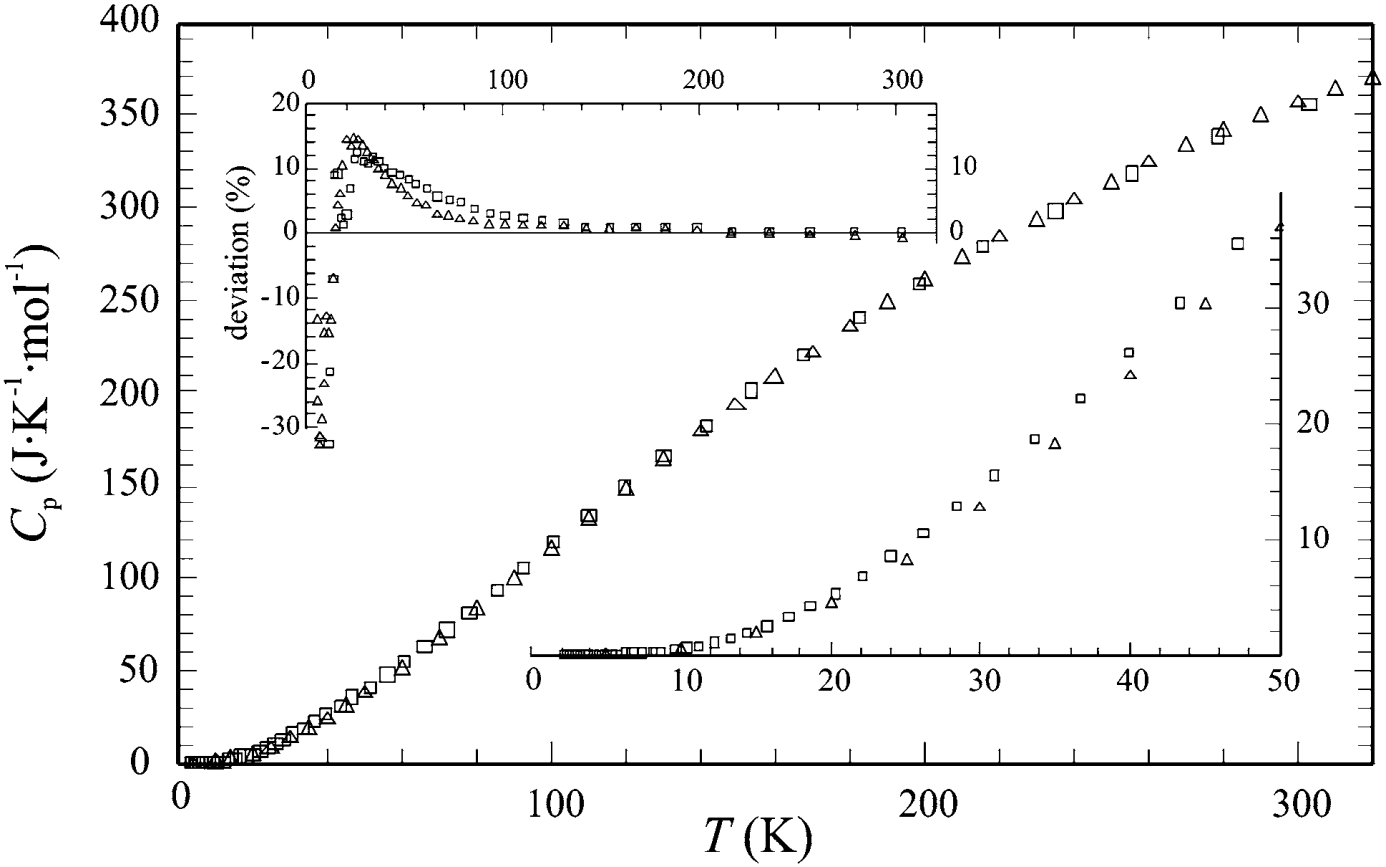
PPMS-measured molar heat capacities of phlogopite in the temperature range 0–300 K from this study (sample Phl100, open squares), compared to the *C*_p_ data of Robie and Hemingway (1984), measured using low-*T* adiabatic calorimetry (open triangles). The *T*-range 0–50 K is enlarged in the lower right inset. A deviation plot of the PPMS data to the DFT-derived *C*_p_ of Phl of this study and to the Robie and Hemingway *C*_p_ is shown in the upper left, i.e., 100(*C*_p_^PPMS^ − *C*_p_^DFT^)/*C*_p_^DFT.^(open squares) and 100(*C*_p_^PPMS^ − *C*_p_^lit.^)/*C*_p_^lit.^(open triangles)

PPMS-measured heat capacities of Phl100 are shown in Fig. 1, where they are compared to *C*_p_ data measured by Robie and Hemingway (1984) on a natural near-phlogopite mica (Burgess, Ontario) using low-TAC. This natural Phl deviated from the ideal end-member formula by the presence of 1–3 wt% FeO, TiO_2_ and F, and Robie and Hemingway (1984) have applied a correction procedure to account for these impurities. A deviation plot of the two *C*_p_ data sets, i.e. 100(*C*_p_^PPMS^ − *C*_p_^literature^)/*C*_p_^literature^, where *C*_p_^literature^ is the corrected data set, is shown as an inset to Fig. 1. At *T*’s between ~15 K and 200 K, the PPMS-measured *C*_p_ data of the synthetic samples are somewhat larger than the adiabatic ones reaching a maximum of ~15% deviation at 20 K, whereas PPMS measured *C*_p_ is slightly lower by 0.8% at room temperature. The lowest temperature segment < 15 K, where absolute *C*_p_ values are < 2 J/(mol K), is characterised by negative deviations of a few tens of a percent.

Following the methods described in Benisek and Dachs (2018), we have also computed phlogopite’s low-*T* heat capacity using DFT. To avoid symbol overlap, the data have not been plotted in the main Fig. 1, but their deviation to the PPMS data is also shown in the inset to Fig. 1. At ambient *T*, the agreement between PPMS-measured and DFT-derived *C*_p_ is very good (deviation < 0.3%); at lower *T*’s, the deviation trend is largely similar to that observed for the adiabatic data.

Based on Eq. (1), we have computed a value of *S*_cal_ = 319.4 ± 2.2 J/(mol K) from our *C*_p_ data measured on synthetic pure phlogopite Phl100. This value represents the vibrational entropy and assuming Al-avoidance in the tetrahedral sheet of phlogopite (e.g., Holland and Powell 1990, 1998), implying the end-member formula KMg_3_[(OH)_2_(AlSi)^T1^(Si_2_)^T2^O_10_], i.e., splitting the four tetrahedral sites into T1 and T2, a configurational entropy *S*_cfg_ = − 2R(2·0.5ln(0.5)) = 11.53 J/(mol K) has to be added to *S*_cal_. This results in a final standard entropy *S*^o^ = 330.9 ± 2.2 J/(mol K) for phlogopite. Its *S*_cal_ is larger by ~1% than the corresponding value of *S*_cal_ = 315.9 ± 1.0 J/(mol K) as given by Robie and Hemingway (1984). The reasons for that are discussed below. A similar value of *S*_cal_ = 315.7 J/(mol K) was obtained from our DFT-calculated low-*T* heat capacities.

##### Eastonite

It is not possible to derive Eas’s heat capacity and thus vibrational entropy directly via calorimetry, because this phase does not exist physically. We can, however, compute Eas’s heat capacity via DFT in a similar manner as done for Phl. In case of Phl, the DFT calculation resulted in a *S*_cal_ value that agreed within ~1% with the calorimetric value. The same level of accuracy can also be expected for an analogous DFT calculation on Eas. The Eas end-member is even easier to handle computationally, because T1 is completely filled with Al. *S*_cal_ = 294.5 ± 3.0 J/(mol K) for Eas was derived in this manner from DFT-computed heat capacities at constant volume converting them into *C*_p_ following Benisek and Dachs (2018) and using Eq. (1). For completely ordered Eas this value is identical to its standard entropy *S*^o^.

#### Super-ambient heat capacity

##### Phlogopite

DSC-measured heat capacities on sample Phl100 are plotted in Fig. S2a as function of temperature, together with data measured by Robie and Hemingway (1984) on natural Burgess-phlogopite applying the same calorimetric method. Additionally shown is CASTEP-calculated *C*_p_. The PPMS data (two data < 300 K), as well as the CASTEP-C_p,_ agree well with the DSC data of this study around ambient *T* (0.2% deviation). For the CASTEP-derived *C*_p_ this agreement holds up to 580 K, whereas *C*_p_ data from the uppermost two DSC series become increasingly lower by up to 3% compared to CASTEP calculated *C*_p_.

Fitting our lowermost three DSC data series, combined with the CASTEP *C*_p_ data, to Eq. (2), yields the following heat capacity polynomial for Phl (in J/(mol K), uncertainty represents 1*σ*):

5 $$\begin{aligned} C_{\text{p}} & = 667.37\left( { \pm 7} \right) - 3914.50\left( { \pm 258} \right) \cdot T^{ - 0.5} - 1.52396\left( { \pm 0.15} \right)\\ & \quad \times 10^{7} \cdot T^{ - 2} + \, 2.17269\left( { \pm 0.25} \right) \times 10^{9} \cdot T^{ - 3} . \\ \end{aligned}$$

Equation (5) reproduces DSC-measured/CASTEP-calculated *C*_p_ to within 0.2 ± 0.1%.

##### Eastonite

The *C*_p_ behaviour of Eas above 298 K, based on our DFT calculations, is given by the polynomial [in J/(mol K), uncertainty represents 1*σ*]:

6 $$\begin{aligned} C_{\text{p}} & = 656.91\left( { \pm 14} \right) - 3622.01\left( { \pm \,503} \right)\, \cdot \,T^{ - 0.5} - 1.70983\left( { \pm \,0.33} \right)\\ & \quad \times 10^{7} \,\cdot\,T^{ - 2} + 2.31802\left( { \pm \,0.59} \right) \times 10^{9} \, \cdot \,T^{ - 3} . \\ \end{aligned}$$

Phl has a higher molar heat capacity than Eas below 1150 K.

#### Standard enthalpy of formation of phlogopite and eastonite

Because our calorimetrically derived *S*^o^ = 330.9 ± 2.2 J/(mol K) for Phl is larger by 1–1.5% than that used, e.g. in the data base of Holland and Powell (2011) or given by Berman et al. (2007) (*S*^o^ = 326 J/(mol K) and *S*^o^ = 327.26 J/(mol K), respectively), we have re-evaluated experimental data on the stability of Phl + Qz based on the reaction

7 $$\begin{aligned} 2\;{\text{Phlogopite}} + 6\;{\text{quartz}} & = 3\;{\text{enstatite}} + 2\;{\text{sanidine}} \\ & \quad + 2\;{\text{H}}_{2} {\text{O}}, \\ \end{aligned}$$

in order to see the effect of this revised entropy value of Phl on its enthalpy of formation value, $\Delta H^{\text{o}}_{\text{f}}$. For that purpose we used 13 experimental brackets determined on reaction (7) either in a pure H_2_O fluid or in fluids with reduced H_2_O activity (H_2_O–CO_2_ or H_2_O–KCl fluids) obtained by Bohlen et al. (1983), Clemens (1995), Aranovich and Newton (1998) and Berman et al. (2007). The evaluation of these experimental data (compiled in Table S2) yields $\Delta H^{\text{o}}_{{{\text{f}},{\text{Phl}}}}$ = − 6209.83 ± 1.10 kJ/mol, and we use this value, *S*^o^ = 330.9 ± 2.2 J/(mol K) and Eq. (5), for *C*_p_ in any further computations below involving Phl.

Benisek and Dachs (2018) have calculated standard enthalpies of formation values, $\Delta H^{\text{o}}_{\text{f}}$, for a number of rock-forming end-members based on DFT calculations. Their result for Phl ($\Delta H^{\text{o}}_{{{\text{f}},{\text{Phl}}}}$ = − 6207.55 kJ/mol) is close to the phase-equilibrium derived value presented above based on phlogopite’s revised *S*_o_ from this study. For end-member Eas, a similar DFT calculation yields $\Delta H^{\text{o}}_{\text{f}}$ = − 6360.5 kJ/mol, 30–35 kJ more negative than values given in Berman et al. (2007) or appearing in Holland and Powell (2011).

#### Volume

**Fig. 2:**
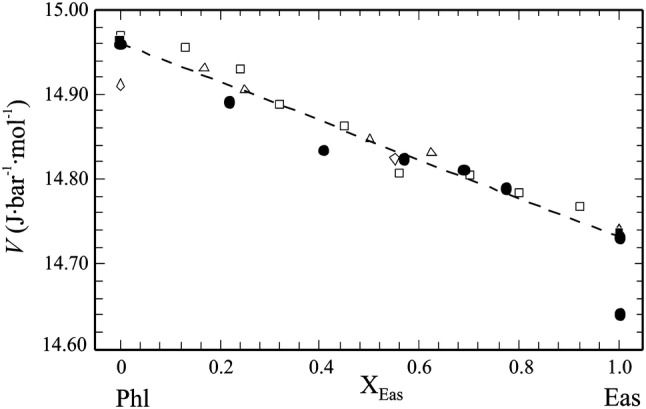
Molar volumes of synthetic members of the Phl–Eas binary of this study (dots; the values for ordered and disordered Eas stem from DFT calculations, all others from X-Ray powder patterns), compared to literature values. Open triangles: Hewitt and Wones (1975), Open squares: Circone and Navrostky (1992), open diamonds: Berman et al. (2007), filled squares: Holland and Powell (2011). The broken line represents ideal mixing

The molar volumes, *V*^o^, of the end-members Phl100 and Eas100, as well as of all solid solution members are listed in Table 2 and are shown in Fig. 2 as function of *X*_Eas_. Our value of 14.958 ± 0.002 J/(mol bar) for *V*^o^ of Phl100 agrees closely with published values. The DFT-computed *V*^o^ = 14.732 J/(mol bar) for disordered Eas, converting the calculated volume at 0 K to 298.15 K using a method outlined in Benisek and Dachs (2018), is identical to that given by Circone et al. (1991). The DFT-calculated *V*^o^ of ordered Eas is, on the other hand, somewhat smaller (14. 647 J/(mol bar)).

**Table 2:** Lattice parameters and volumes of synthetic members of the Phl–Eas join

	*X*_Eas_/(*X*_Eas_+*X*_Phl_)	*a* (Å)	*b* (Å)	*c* (Å)	*β* (°)	Volume (Å)	Volume (J/mol·bar)
Phl100	0	5.3146	9.2038	10.3089	99.883	496.77 (8)	14.958 (2)
Phl80Eas20	0.22	5.3032	9.1894	10.3009	99.886	494.54 (8)	14.891 (2)
Phl60Eas40	0.41	5.2952	9.1711	10.2963	99.864	492.63 (8)	14.833 (2)
Phl40Eas60	0.57	5.2901	9.1676	10.3028	99.866	492.26 (8)	14.822 (2)
Phl20Eas80	0.69	5.2860	9.1627	10.3078	99.870	491.86 (8)	14.810 (2)
Eas100	0.77	5.2787	9.1603	10.3107	99.900	491.15 (8)	14.789 (2)
Eas-ord.	1						14.647^a^
Eas-disord.	1						14.732^a^

^a^Molar volume of ordered and disordered eastonite as derived from DFT computations

### A new activity model for Mg–Al biotites based on Mg–Al ordering

In the following, we present a new activity model for Mg–Al biotites that has been formulated introducing Mg–Al order–disorder. It is based on results from DFT calculations (Table 3) showing that an enthalpy of disordering of Δ*H*_dis_ = 34.5 ± 3 kJ/mol is associated with the disordering of Mg and Al on the M sites of Eas:

8 $$\begin{array}{*{20}c} {\text{ordered - Eas}} & = & {\text{disordered - Eas}} \\ {{\text{KAl}}^{\text{M1}} \left( {{\text{Mg}}_{ 2} } \right)^{\text{M2}} \left[ {\left( {\text{OH}} \right)_{ 2} \left( {{\text{Al}}_{ 2} } \right)^{\text{T1}} \left( {{\text{Si}}_{ 2} } \right)^{\text{T2}} {\text{O}}_{ 10} } \right]} & = & {{\text{K}}\left( {{\text{Al}}_{ 1/ 3} {\text{Mg}}_{ 2/ 3} } \right)^{\text{M1}} \left( {{\text{Al}}_{ 2/ 3} {\text{Mg}}_{ 4/ 3} } \right)^{\text{M2}} \left[ {\left( {\text{OH}} \right)_{ 2} \left( {{\text{Al}}_{ 2} } \right)^{\text{T1}} \left( {{\text{Si}}_{ 2} } \right)^{\text{T2}} {\text{O}}_{ 10} } \right]} \\ \end{array}$$

**Table 3:** CASTEP-energies (Δ*U*^0K^) of completely ordered eastonite (Eas, *Q* = 1), for completely disordered eastonite (dEas, *Q* = 0) and two intermediate ordered eastonites (Eas-1, Eas-2)

	*Q* ^a^	Octahedral site fraction of Al	Δ*U*^0K^ (kJ/mol)	Figure in suppl. materials
Eas	1	*X*_Al_^M1^ = 1, *X*_Al_^M2^=0	− 925,452.7 ± 1.4	Fig. S3
Eas-1	0.625	*X*_Al_^M1^ = 3/4, *X*_Al_^M2^=1/8	− 925,437.9 ± 3.0	
Eas-2	0.250	*X*_Al_^M1^ = 1/2, *X*_Al_^M2^=1/4	− 925,428.3 ± 3.0	
dEas	0	*X*_Al_^M1^ = 1/3, *X*_Al_^M2^=1/3	− 925,418.2 ± 2.4	Fig. S4

The enthalpy difference between Eas and dEas gives the enthalpy of disordering, Δ*H*_dis_ = 34.5 kJ/mol

^a^*Q* = *X*_Al_^M1^ − *X*_Al_^M2^

Note that ‘Eas’ is used in the following for ordered eastonite and ‘dEas’ for disordered eastonite. The third end-member in this model is Phl (site distributions of the end-members are given in Table 4). Similar to published biotite activity models (e.g. Holland and Powell 1998; Powell and Holland 1999; White et al. 2000, 2007, 2014; Holland and Powell 2006; Tajčmanová et al. 2009) tetrahedral sites have been split into T1 and T2 sites to guarantee Al-avoidance.

**Table 4:** End-members and site distributions for Mg-Al biotite

End-member	Formula	Site distribution			
		M1	M2	T1	T2
Phl	KMg_3_[(OH)_2_(AlSi_3_)O_10_]	Mg	Mg_2_	AlSi	Si_2_
Eas (ordered)	K(AlMg_2_)[(OH)_2_(Al_2_Si_2_)O_10_]	Al	Mg_2_	Al_2_	Si_2_
dEas (disordered)	K(AlMg_2_)[(OH)_2_(Al_2_Si_2_)O_10_]	Al_1/3_Mg_2/3_	Al_2/3_Mg_4/3_	Al_2_	Si_2_

Mg–Al ordering on the octahedral sites can then be described by the ordering parameter

9 $$Q \equiv X_{\text{Mg}}^{{{\text{M}}2}} - X_{\text{Mg}}^{{{\text{M}}1}} = X_{\text{Al}}^{{{\text{M}}1}} {-} \, X_{\text{Al}}^{{{\text{M}}2}} ,$$

where *X*_Mg_^M2^ and *X*_Mg_^M1^ are the site fractions of Mg on M1 and M2, respectively. As bulk-composition parameter octahedral Al (Al^VI^) is introduced to describe the compositional variation along the Phl–Eas join.

For the internal reaction (8), equilibrium is given by the following relation:

10 $$\begin{aligned} \Delta G_{dis} & = 0 \\ & = \Delta H_{\text{dis }} + 2QW_{{{\text{Eas}},{\text{dEas}}}} - W_{{{\text{Phl}},{\text{Eas}}}} + W_{\text{Phl,dEas}} + {\text{Al}}^{\text{VI}} \left( {W_{\text{Phl,Eas}} - W_{{{\text{Phl}},{\text{dEas}}}} - W_{{{\text{Eas}},{\text{dEas}}}} } \right) \\ & \quad + RT\ln \left[ {\frac{{\left( {3 - {\text{Al}}^{\text{VI}} - 2Q} \right)\left( {{\text{Al}}^{\text{VI}} - Q} \right)}}{{\left( {3 - {\text{Al}}^{\text{VI}} + Q} \right)\left( {{\text{Al}}^{\text{VI}} + 2Q} \right)}}} \right]^{{\frac{2}{3}}} , \\ \end{aligned}$$

where Δ*H*_dis_ is the enthalpy of disordering according to Eq. (8), and *W*_Phl,Eas_, *W*_Phl,dEas_ and *W*_Eas,dEas_ are binary macroscopic symmetric interaction parameters (e.g., Ganguly 2008). The derivation of Eq. (10) is given in the supplementary materials. For known values of Δ*H*_dis_ and the *W*_i_’s, eq. (10) can then be solved for the equilibrium degree of order, *Q*_eq_, for given values of *T* and Al^VI^ in biotite.

### Calibrating the Mg–Al biotite mixing properties

We determine the Mg–Al biotite mixing properties applying an integrated approach that combines results from low-temperature calorimetry, from DFT calculations, from line-broadening in IR spectra and from evaluating existing phase-equilibrium data.

#### Vibrational excess entropies of mixing

**Fig. 3:**
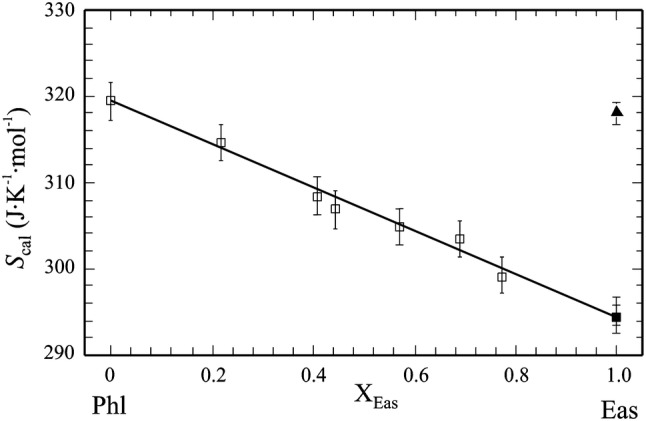
Calorimetric entropies, *S*_cal_, at 298.15 K of synthetic members of the Phl–Eas binary (open squares), derived from PPMS measurements (data from Table 1). The extrapolated standard entropy of eastonite (*S*^o^ = 294.0 ± 3.7 J/(mol K)) is shown as filled square. It is identical to DFT-calculated *S*^o^ (294.5 ± 3.0 J/(mol K)). The data indicate ideal vibrational-entropic mixing along the Phl–Eas binary. Literature estimates of *S*^o^ of eastonite are shown as filled triangle. Error bars are ± 2*σ*

The calorimetric entropies *S*_cal_ of the studied Mg–Al biotites at 298.15 K are plotted in Fig. 3 as function of composition (the data are listed in Table 1). Using our calorimetrically determined value of 319.4 ± 2.2 J/(mol K) for the vibrational entropy of Phl and the DFT-computed *S*^o^ of Eas of 294.5 ± 3.0 J/(mol K) at 298.15 K, all solid-solution members fall on the line defining ideal mixing within error. If, on the other hand, a value for *S*^o^ of Eas was determined by linear extrapolation, i.e. fitting exclusively the calorimetric data starting from Phl up to Phl20Eas80, a value of 294.0 ± 3.7 J/(mol K) would result. This value agrees excellently with the DFT-calculated *S*^o^ value and thus confirms that our DFT-calculations for the Eas end-member yield reliable results. Estimates of *S*^o^ of end-member Eas, appearing in the literature, are considerably larger by ~8% and need to be corrected [317.4 ± 1.2 J/(mol K); Circone and Navrotsky 1992, 318 J/(mol K); Holland and Powell 2011, 318.59 J/(mol K); Berman et al. 2007]. In summary, our calorimetric data along the Phl-Eas join indicate that there are no vibrational excess entropies of mixing in this binary. The enthalpic mixing parameters of this join have thus no temperature dependence, i.e. the Phl–Eas solid-solution can be treated as a regular solution with *W*_G,ij_ = *W*_H,ij_, e.g. *W*_G,Phl,Eas_ = *W*_H,Phl,Eas_. For simplicity, we drop the ‘H’ subscript in the following, so that *W*_H,Phl,Eas_ ≡ *W*_Phl,Eas_. Our finding that the vibrational entropy of the Mg–Al biotites behaves ideal is similar to another Tschermak substituted binary, the diopside–CaTs pyroxenes, where ideal vibrational mixing was obtained as well (Etzel et al. 2007).

#### Excess enthalpies of mixing: Δ*H*_ex_ and *W*_Phl,Eas_

##### Δ*H*_ex_ from DFT calculations (Δ*H*_ex_^DFT^)

**Fig. 4:**
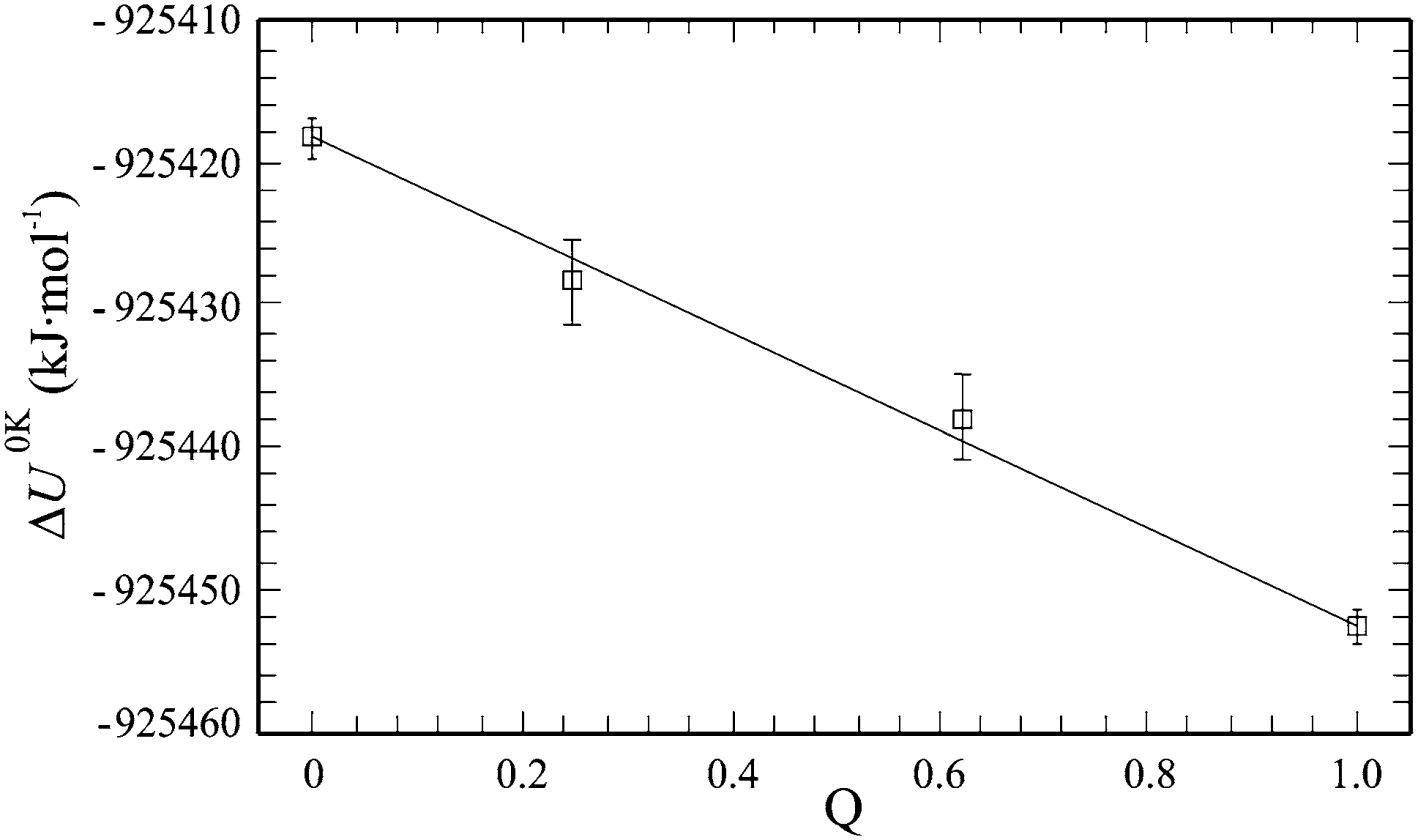
CASTEP-energies (Δ*U*^0K^) as function of the order parameter *Q*, for completely Mg–Al ordered eastonite (Eas, *Q* = 1), for completely disordered eastonite (dEas, *Q* = 0) and two intermediate ordered eastonites (data from Table 3). Error bars are ± 2*σ*

*Binary join ordered eastonite* (*Eas*)*–disordered eastonite* (*dEas*) Figure 4 is a plot of CASTEP-energies at 0 K (Δ*U*^0K^), listed in Table 3, calculated for completely ordered Eas (all Al^VI^ on M1), disordered Eas (one Al^VI^ distributed over M1 and M2 so that *X*_Al_^M1^ = *X*_Al_^M2^ = 1/3), and two partly (dis-)ordered configurations as function of the order parameter *Q*. For completely ordered Eas, $\Delta U^{{0{\text{K}}}}_{\text{Eas}}$ = − 925452.7 kJ/mol. In order to determine Δ*U*^0K^ for disordered Eas (*X*_Al_^M1^ = *X*_Al_^M2^=1/3), we have computed CASTEP energies for four different configurations distributing Mg–Al over the three M sites (fully ordered and disordered structures are shown in the supplementary materials, Figs. S3, S4). The mean gives $\Delta U^{{0{\text{K}}}}_{\text{Eas - disord}}$ = − 925,418.2 kJ/mol (Table 3). The uncertainty of this value is ± 2.4 kJ/mol, due to the spread of the data resulting from the different octahedral Mg–Al configurations.

As the energies calculated for the two intermediate (dis-)ordered states fall on the line representing ideal mixing within error (Fig. 4), *W*_Eas,dEas_ ~ 0 and can be set to zero in Eq. (10). A similar ideal mixing behaviour was observed for MgAl_2_O_4_–Al_8/3_O_4_ spinels, which could be described to an excellent approximation with a constant value of the Gibbs energy of the ordering reaction alone (Sack 2014). The enthalpy of disordering of Eas is given by the difference: Δ*H*_dis_ = $\Delta U^{{0{\text{K}}}}_{\text{dEas}}$ − $\Delta U^{{0{\text{K}}}}_{\text{Eas}}$ = 34.5 kJ/mol with an uncertainty of ca. ± 3 kJ/mol.

*Binary join phlogopite* (*Phl*)*–ordered eastonite* (*Eas*). In order to determine *W*_Phl,Eas_ and *W*_Phl,dEas_ from DFT calculations, we have applied the single-point defect method (e.g., Sluiter and Kawazoe 2002). In the case of the ordered Eas end-member, this means that one Mg^2+^ defect atom is introduced into the octahedral sheet, replacing one Al^3+^ atom there, coupled with a Si^4+^ defect replacing one adjacent tetrahedral Al^3+^. For maintaining local charge balance, the placement of this tetrahedral Si^4+^ defect atom was chosen in such a way that the ${\text{Mg}}^{ 2+ }_{\text{oct-defect}}{-}{\text{Si}}^{ 4+ }_{\text{tet-defect}}$ distance was 3.2 Å in all cases. This is the shortest structurally possible Mg^2+^–Si^4+^ distance and there are exactly four possible configurations of this kind in the Eas end-member. In a cell with *Z* = 12 this means that *X*_Eas_ = 11/12 = 0.917. Similar ‘inverse Tschermak-defects’ were incorporated into ordered Eas cells with *Z* = 8 and *Z* = 6, as well as one double defect in a cell with *Z* = 6. The calculated CASTEP energies for all structures of the Phl–Eas join are given in Table 5. On the Phl-side we have constructed a phlogopite with *Z* = 8 containing one classical ‘Tschermak-defect’, i.e. $\left( {{\text{Al}}^{ 3+ } } \right)^{\text{oct}} \left( {{\text{Al}}^{ 3+ } } \right)^{\text{tet}} \left( {{\text{Mg}}^{ 2+ } } \right)^{\text{oct}}_{ - 1} \left( {{\text{Si}}^{ 4+ } } \right)^{\text{tet}}_{ - 1}$ in a similar manner as described above for ordered Eas. The value given for Δ*U*^0K^ of Phl in Table 5 is the mean of seven separate CASTEP calculations, each with a different tetrahedral Al-Si distribution representing Al-avoidance leading to $\Delta U^{{0{\text{K}}}}_{\text{Phl}}$ = − 1,078, 178.5 ± 2.1 kJ/mol. The uncertainty of ± 2.1 kJ/mol reflects the energetic response of the structure to this variation in the tetrahedral layer. The excess enthalpy, Δ*H*_ex_, of the solid-solution members was then calculated from:

11 $$\begin{aligned} \Delta H_{\text{ex}}^{\text{DFT}} & = \Delta U_{\text{ex}} = \Delta U^{{0{\text{K}}}}_{\text{solution}} - \Delta U^{{0{\text{K}}}}_{\text{mech}} \\ & = \Delta U^{{0{\text{K}}}}_{\text{solution}} {-}\left[ {\Delta U^{{0{\text{K}}}}_{\text{Phl}} ( 1- X_{\text{Eas}} ) \, + \, \Delta U^{{0{\text{K}}}}_{\text{Eas}} X_{\text{Eas}} } \right]. \\ \end{aligned}$$

**Table 5:** CASTEP-energies (Δ*U*^0K^) for the binary phlogopite (Phl)–ordered eastonite (Eas)

	*X*_East_	Δ*U*^0K^ (kJ/mol)	Δ*H*_ex_ (kJ/mol)	*W*_Phl,Eas_ (kJ/mol)^a^	Comment
Eas^b^	1	− 925,452.69 ± 1.40	0		End member
Phl	0	− 1,078,178.54 ± 2.10	0		End member
Eas67	2/3	− 976,359.34	1.97	8.85	dd in cell with *Z* = 6
Eas83	5/6	− 950,905.97	1.03	7.36	sd in cell with *Z* = 6
Eas88	7/8	− 944,542.25	1.17	10.70	sd in cell with *Z* = 8
Eas92	11/12	− 938,179.13	0.71	9.35	sd in cell with *Z* = 12
Eas13	1/8	− 1,059,086.78	1.03	9.42	sd in cell with *Z* = 8

Δ*H*_ex_, calculated from Eq. (11) and *W*_Phl,Eas_, calculated from Eq. (12) are also given. The tetrahedral Si-Al distribution is that of Al-avoidance

*sd* single point defect, *dd* double defect

^a^Mean gives *W*_Phl,Eas_ = 9.1 ± 1.2 kJ/mol as appearing in the text and termed *W*_Phl,Eas_^DFT^

^b^Structures used in the CASTEP calculations are shown in Figs. S3 and S4

Based on the relation Δ*H*_ex_^DFT^ = Δ*U*_ex_ + *P*Δ*V*_ex_, the identity Δ*H*_ex_^DFT^ = Δ*U*_ex_ holds, because the *P*Δ*V*_ex_-term is zero (see below).

**Fig. 5:**
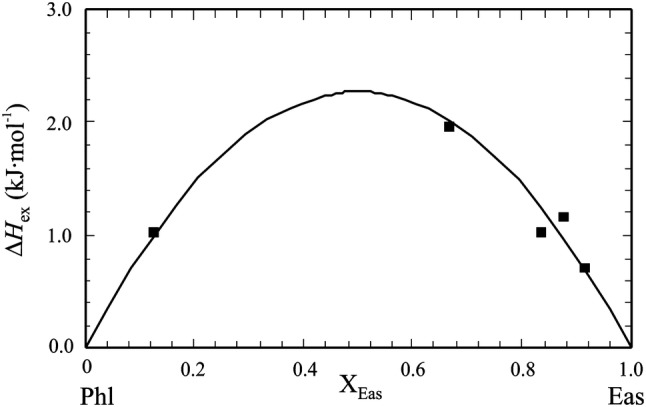
Δ*H*_ex_ of the phlogopite (Phl)–ordered-eastonite (Eas) binary, derived from DFT calculations using cells with single-point or double defects (data from Table 5). Curve represents Δ*H*_ex_ computed from Eq. (11) using a symmetrical *W*_Phl,Eas_ = 9.1 ± 1.2 kJ/mol

The resulting Δ*H*_ex_^DFT^‘s (Table 5) are shown in Fig. 5, plotted vs. *X*_Eas_, and indicate positive deviation from ideality in all cases. Using a symmetrical mixing model, values for *W*_Phl,Eas_^DFT^ can be computed from the general relation:

12 $$W_{{{\text{Phl}},{\text{Eas}}}} = \Delta H_{\text{ex}} /\left[ {( 1- X_{\text{Eas}} )X_{\text{Eas}} } \right].$$

**Fig. 6:**
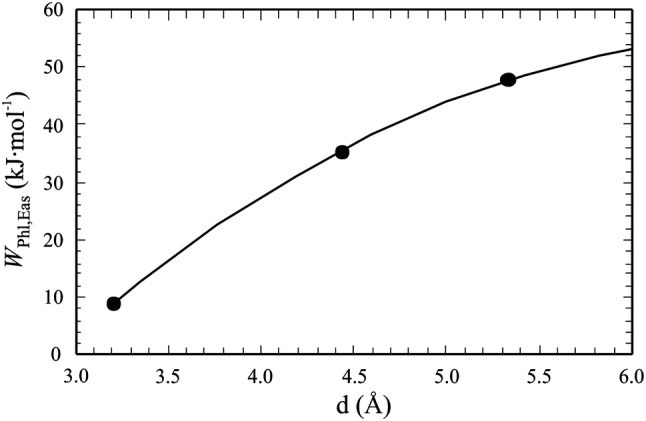
DFT-derived interaction parameter *W*_Phl,Eas_^DFT^ as function of the distance *d* between the octahedral and the tetrahedral defect

The mean value and standard deviation of these data give *W*_Phl,Eas_^DFT^ = 9.1 ± 1.2 kJ/mol and a positive Δ*H*_ex_^DFT^ of at maximum 2.3 kJ/mol for the Phl–Eas join. This *W*_Phl,Eas_^DFT^ represents the structural situation with the shortest possible ${\text{Mg}}^{ 2+ }_{\text{oct-defect}} {{{-}Si}}^{ 4+ }_{\text{tet-defect}}$ distance of 3.2 Å (four different realisations). The corresponding extent of non-ideality should be considered as minimum non-ideality along the Phl–Eas join and would apply for biotites with the *‘*strictest’ local charge balance (LCB) in their structures. The placement of the Si^4+^ defect atom in the ordered structure of Eas can, however, be done in two more ways, with larger distances of 4.43 Å and 5.33 Å away from the octahedral Mg^2+^ defect atom. We have performed similar DFT calculations with these 2nd and 3rd next nearest Mg^2+^–Si^4+^ distances. The resulting larger *W*_Phl,Eas_^DFT^’s are listed in Table 6 and the correlation between Mg^2+^–Si^4+^ distance, *d* (in Å), and these *W*‘s is shown in Fig. 6. It is given by:

13 $$W_{{{\text{Phl}},{\text{Eas}}}}^{\text{DFT}} \left( {{\text{kJ}}/{\text{mol}}} \right) = - 1 1 2. 3+ 4 9. 8\, \cdot \,d{-} 3. 7\, \cdot \,d^{ 2} .$$

**Table 6:** CASTEP-energies (Δ*U*^0K^) of the binary phlogopite (Phl)–ordered eastonite (Eas) as a function of the distance *d* between the octahedral and the tetrahedral defect

	*d* (Å)	*X*_Eas_	Δ*U*^0K^ (kJ/mol)	Δ*H*_ex_ (kJ/mol)	*W*_Phl,Eas_ (kJ/mol)	Comment
Eas		1	− 925,452.69	0		End-member
Phl		0	− 1,078,178.28	0		end-member
Eas92	3.20^a^	11/12	− 938,179.13	0.69	9.06	sd in cell with Z = 12
Eas92	4.43^b^	11/12	− 938,177.10	2.72	35.58	sd in cell with Z = 12
Eas92	5.33^c^	11/12	− 938,176.16	3.66	47.90	sd in cell with Z = 12

Δ*H*_ex_, calculated from Eq. (11) and *W*_Phl,Eas_, calculated from Eq. (12) are also given

*sd* single point defect, *dd* double defect

^a^1st, ^b^2nd, ^c^3rd next nearest distance

##### Δ*H*_ex_ from line broadening of IR spectra (Δ*H*_ex_^Δcorr^)

**Fig. 7:**
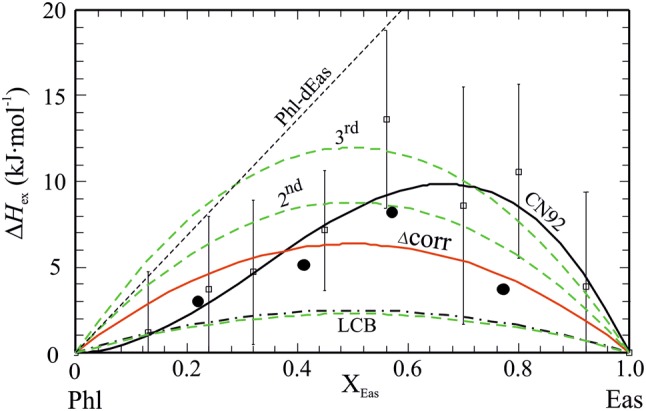
Excess enthalpies of mixing, Δ*H*_ex_, along the Phl–ordered Eas binary. Experimental high-temperature solution calorimetry data of Circone and Navrotsky (1992) are shown as open squares (with error bars of ± 2*σ*), the asymmetric curve is their fit to their data. Dots is Δ*H*_ex_ derived from line broadening in IR spectra (data from Table 1, mid wave number region), the curve labelled ‘Δcorr’ is a symmetric fit to these data yielding *W*_PhlEas_ = 25.4 kJ/mol. Curves labelled ‘LCB’, ‘2nd’ and ‘3rd’ are Δ*H*_ex_, based on DFT-calculations, in which defect-atom pairs in the biotite structure have 1st (lowest curve representing strict local charge balance, LCB), 2nd and 3rd next nearest distances to each other (Table 6). Dot-dashed curve is Δ*H*_ex_ as used in published biotite activity models, i.e., *W*_PhlEas_ = 10 kJ/mol (e.g., Holland and Powell 2006; White et al. 2000, 2007, 2014; Tajčmanová et al. 2009). Ideal mixing for the binary Phl–disordered Eas (dEas) is shown as dashed straight line

The line-broadening of IR spectra as result of solid-solution formation has been established as a method to determine qualitatively (e.g., Boffa Ballaran et al. 1999; Carpenter and Boffa Ballaran 2001) and quantitatively (e.g., Dachs et al. 2014) the enthalpic mixing behaviour of solid-solutions. The IR spectra in the mid wave number region 400–600 cm^−1^ are shown in Fig. S5 for Phl100, Phl80Eas20, Phl60Eas40, Phl40Eas60 and Eas100. The peak in the wave number range 460–500 cm^−1^ appearing in all spectra was subject to autocorrelation analysis (Salje et al. 2000) using a self-written Mathematica program. The resulting Δcorr-values are given in Table 1 and were fitted to the following equation:

14 $$\delta \Delta {\text{corr}} = \Delta {\text{corr}}_{\text{Phl}} \left( { 1- X_{\text{Eas}} } \right) + \Delta {\text{corr}}_{\text{Eas}} X_{\text{Eas}} + \, \left( { 1- X_{\text{Eas}} } \right)X_{\text{Eas}} W^{{\Delta {\text{corr}}}} ,$$

with the two unknowns Δcorr_Eas_ and *W*^Δcorr^, which corresponds to a symmetrical interaction parameter. Their values are: Δcorr_Eas_ = 37.1 ± 0.7 and *W*^Δcorr^ = 86.0 ± 1.4. The line-broadenings in the IR spectra of the Phl–Eas binary thus indicate positive deviations from ideality characterised by a δΔcorr value of 21.5 ± 0.3 (δΔcorr = *W*^Δcorr^/4). This δΔcorr gives the maximal deviation from ideality in terms of Δcorr. In order to translate the δΔcorr into corresponding Δ*H*_ex_^Δcorr^ values, we used the correlation between the two established by Etzel and Benisek (2008):

15 $$\Delta H_{\text{ex}}^{\Delta\text{corr}} = \left( {d + k\,{\text{norm}}V^{\text{exc}}_{\text{int}} } \right)n,$$

with *n* = 22 (number of atoms per formula unit). The values *d* = 13.4 J cm mol^−1^ and *k* = 308 J cm^−2^, valid for the intermediate wave number region, were taken from Etzel and Benisek (2008). The integrated excess volume of mixing normalised to one atom per formula unit (${\text{norm}}V^{\text{exc}}_{\text{int}}$) is zero for this binary (see below). The values are given in Table 1 and are plotted in Fig. 7. This results in a maximum Δ*H*_ex_^Δcorr^ of 6.4 kJ/mol, giving a symmetrical interaction parameter *W*_PhlEas_^Δcorr^ = 25.4 kJ/mol for the Phl–Eas join. Evaluating the line broadening of IR spectra from the high wave number region of 790–1330 cm^−1^ yields a similar positive Δ*H*_ex_^Δcorr^, which is, however, smaller (Table 1) and would give a *W*_PhlEas_^Δcorr^ = 10.3 kJ/mol.

##### *W*_Phl,Eas_ and $H^{\text{o}}_{{{\text{f}},{\text{Eas}}}}$ from evaluating phase-equilibrium data

In their experimental study on the stability of Mg–Fe–Al biotite, Berman et al. (2007) determined the Al-solubility in Mg–Al biotite from reversed phase-equilibrium data. For the reaction:

16 $$\begin{aligned} 3\;{\text{Eastonite}} + 6\;{\text{quartz}} & = 2\;{\text{phlogopite}} + 3\;{\text{sillimanite}} \\ & \quad + {\text{sanidine}} + {\text{H}}_{2} {\text{O,}} \\ \end{aligned}$$

they determined four composition-brackets over the *P*–*T* range ~650–750 °C and 1.1–3.2 kbar which define the Al-saturation level of biotite to a value of 1.60 ± 0.04 in the assemblage (Mg–Al)-biotite-sillimanite-sanidine-quartz under the presence of water starting from mixes with different Al-contents in biotite. The equilibrium relation for this case can be rearranged to

17 $$\frac{{\Delta H_{\text{R}}^{\prime } + \mathop \smallint \nolimits_{298.15}^{T} \Delta C_{{{\text{p}},{\text{R}}}} {\text{d}}T - T\left( {\Delta S_{\text{R}}^{\text{o}} + \mathop \smallint \nolimits_{298.15}^{T} \frac{{\Delta C_{{{\text{p}},{\text{R}}}} }}{T}{\text{d}}T} \right) + \mathop \smallint \nolimits_{1}^{P} \Delta V_{\text{R}}^{\text{o}} {\text{d}}P}}{3} = H_{f,Eas}^{o} - \frac{{RT\left( {2\ln a_{\text{Phl}} - 3\ln a_{\text{Eas}} } \right)}}{3},$$

where $\Delta H^{\prime }_{\text{R}} = { 2}\Delta H^{\text{o}}_{{{\text{f}},{\text{Phl}}}} + { 3}\Delta H^{\text{o}}_{{{\text{f}},{\text{Sil}}}} + \Delta H^{\text{o}}_{{{\text{f}},{\text{San}}}} + { 2}\Delta H^{\text{o}}_{{{\text{f}},{\text{H2O}}}} - 6\Delta H^{\text{o}}_{{{\text{f}},{\text{Qz}}}} \cdot \Delta S^{\text{o}}_{\text{R}} \;{\text{and}}\;\Delta V^{\text{o}}_{\text{R}}$ are the entropy- and volume change at standard conditions, Δ*C*_p,R_ is the change in heat capacities. The left-hand side of Eq. (17) was computed from known thermodynamic data ($S^{\text{o}}_{\text{Phl}}$, $\Delta H^{\text{o}}_{{{\text{f}},{\text{Phl}}}}$, *C*_p,Phl_, $S^{\text{o}}_{\text{Eas}}$, *C*_p,Eas_ from this study, all other thermodynamic data and functions from Holland and Powell 2011). The activity term on the right-hand side of Eq. (17) contains the interaction parameters *W*_Phl,Eas_ and *W*_Phl,dEas_ as unknowns in the activity model. Based on Eq. (17) and setting *W*_Phl,dEas_ = 0 kJ/mol (the justification for this choice is given below), optimised values of $\Delta H^{\text{o}}_{{{\text{f}},{\text{Eas}}}}$ and *W*_Phl,Eas_ were then determined from the phase-equilibrium data using the Mathematica function ‘FindMinimum’. This gives $\Delta H^{\text{o}}_{{{\text{f}},{\text{Eas}}}}$ = − 6362.7 kJ/mol and *W*_Phl,Eas_ = 29.8 kJ/mol. We note, however, that these data are uncertain due to the small temperature range of the experiments and other combinations of $\Delta H^{\text{o}}_{{{\text{f}},{\text{Eas}}}}$ and *W*_Phl,Eas_ would equally well reproduce the experimentally determined Al-saturation level of biotite. For that reason we use the experimental data to establish a correlation between $\Delta H^{\text{o}}_{{{\text{f}},{\text{Eas}}}}$ and *W*_Phl,Eas_ by solving Eq. (17) for $\Delta H^{\text{o}}_{{{\text{f}},{\text{Eas}}}}$ and inserting predefined values of the interaction parameter *W*_Phl,Eas_, ranging between 10 and 30 kJ/mol. This results in the following quadratic equation:

18 $$\begin{aligned} \Delta H_{{{\text{f}},{\text{Eas}}}}^{{\text{o}}} \left( {{\text{kJ}}/{\text{mol}}} \right) & = - 6348.5 - 0.34{\mkern 1mu} \cdot {\mkern 1mu} W_{{{\text{Phl}},{\text{Eas}}}} \\ & \quad - 0.002{\mkern 1mu} \cdot {\mkern 1mu} W_{{{\text{Phl}},{\text{Eas}}}}^{2} , \\ \end{aligned}$$

where *W*_Phl,Eas_ is in kJ/mol. Pinning *W*_Phl,Eas_ to the δΔcorr value of 25.4 kJ/mol (mid wave number region), gives $\Delta H^{\text{o}}_{{{\text{f}},{\text{Eas}}}}$ = − 6358.5 ± 1.4 kJ/mol, in good agreement with $\Delta H^{\text{o}}_{{{\text{f}},{\text{Eas}}}}$ derived independently from DFT (− 6360.5 kJ/mol). Using, alternatively, *W*_PhlEas_^Δcorr^ = 10.3 kJ/mol (high wave number region), gives $\Delta H^{\text{o}}_{{{\text{f}},{\text{Eas}}}}$ = − 6352.0 ± 1.4 kJ/mol.

#### Excess volumes of mixing Δ*V*_ex_

The molar volumes of our synthetic Mg–Al biotites (Table 2) are displayed in Fig. 2. Despite some scatter, their variation with *X*_Eas_ follows a linear trend between *V*^o^ of disordered Phl (14.96 J/mol·bar) and disordered Eas (14.73 J/mol·bar, DFT calculated). There are thus no significant excess volumes of mixing along the Phl–Eas join. The DFT-calculated *V*^o^ of ordered Eas is somewhat lower (14.65 J/mol·bar).

## Discussion

### End-member thermodynamic data

#### Phlogopite

In our study we calorimetrically derived a vibrational entropy *S*_cal_ = 319.4 ± 2.2 J/(mol K) at 298.15 K for synthetic pure Phl and, including a *S*_conf_ = 11.53 J/(mol K), we obtained its revised standard entropy as *S*^o^ = 330.9 ± 2.2 J/(mol K). Our *S*_cal_ value is to be preferred over that determined by Robie and Hemingway (1984) on a natural near-phlogopite mica, because the latter deviated from the ideal phlogopite formula by the presence of 1–3 wt% FeO, TiO_2_ and F, requiring a *C*_p_-correction for these impurities. Taking the uncorrected *C*_p_ data of Robie and Hemingway would give, on numerical integration, *S*_cal_ = 312.9 ± 1.0 J/(mol K). This value rises to *S*_cal_ = 315.9 ± 1.0 J/(mol K) using their corrected *C*_p_ data. This value is still 3.5 J/(mol K) lower than the PPMS-derived *S*_cal_, showing that the correction procedure applied by Robie and Hemingway was not sufficiently accurate based on the data available at that time. Our *S*^o^ value is 1–1.5% larger than that given in Holland and Powell (2011) (*S*^o^ = 326.0 J/(mol K)) or Berman et al. (2007) (*S*^o^ = 327.3 J/(mol K)), which rely on the Robie and Hemingway vibrational entropy value of Phl adding *S*_cfg_.

From the revised *S*^o^ of Phl from this study we extracted $\Delta H^{\text{o}}_{{{\text{f}},{\text{Phl}}}}$ = − 6209.83 ± 1.1 kJ/mol by evaluating phase-equilibrium data on the stability of Phl + Qtz (reaction (7), Table S2). Published values of $\Delta H^{\text{o}}_{{{\text{f}},{\text{Phl}}}}$, derived from enthalpies of solution data, agree with this revised value within error, i.e. $\Delta H^{\text{o}}_{{{\text{f}},{\text{Phl}}}}$ = − 6214.1 ± 6.1 kJ/mol (Clemens et al. 1987), and $\Delta H^{\text{o}}_{{{\text{f}},{\text{Phl}}}}$ = − 6211.7 ± 5.6 kJ/mol (Circone and Navrotsky 1992). The phase-equilibrium derived $\Delta H^{\text{o}}_{{{\text{f}},{\text{Phl}}}}$ values of Holland and Powell (2011) and Berman et al. (2007) ($\Delta H^{\text{o}}_{{{\text{f}},{\text{Phl}}}}$ = − 6214.95 ± 2.90 kJ/mol, $\Delta H^{\text{o}}_{{{\text{f}},{\text{Phl}}}}$ = − 6215.86 kJ/mol) are 5–6 kJ/mol more negative than our revised $\Delta H^{\text{o}}_{{{\text{f}},{\text{Phl}}}}$. The reason for this difference is that the above mentioned *S*^o^ of Phl based on Robie and Hemingway’s vibrational entropy value was used by Holland and Powell (2011) and Berman et al. (2007) in extracting $\Delta H^{\text{o}}_{{{\text{f}},{\text{Phl}}}}$. If we would re-evaluate the phase-equilibrium data on Phl + Qz stability, given in Table S2, with that standard entropy value (i.e., *S*^o^ = 326 J/(mol K)), we would get a similar $\Delta H^{\text{o}}_{{{\text{f}},{\text{Phl}}}}$ = − 6215.1 kJ/mol.

This shows that a difference of only 1–1.5% in entropy can cause a 5–6 kJ/mol different enthalpy of formation value of a phase. Our new *C*_p_-polynomial for Phl (Eq. 5) was fitted from the lowermost three DSC data series (282 K–564 K), combined with CASTEP-computed *C*_p_ above ambient *T*, whereas the two DSC-series at higher *T*’s were disregarded. As high-*T* DSC data are usually less accurate than intermediate-*T* ones (Benisek et al. 2009), this procedure seems reasonable and should give more reliable heat capacities of phlogopite, especially at high *T*’s around and above 1000 K. At this temperature, the *C*_p_-polynomial of Eq. (5) yields a 1–1.5% larger *C*_p_ for Phl compared to *C*_p_ calculated from published polynomials (Robie and Hemingway 1984; Circone and Navrotsky 1992; Berman et al. 2007; Holland and Powell 2011) (Fig. S2b).

#### Eastonite

An important aspect of our study is to provide a reliable value for the standard entropy of the Eas end-member. This is *S*^o^ = 294.5 ± 3.0 J/(mol K) based on our DFT calculations, in close agreement with the calorimetrically determined value of *S*^o^ = 294.0 ± 3.7 J/(mol K), derived by linear extrapolation from *S*_cal_ of the studied solid-solution members (Fig. 3). Presently used values for *S*^o^ of Eas in the more recent literature are ~ 8% larger (*S*^o^ = 318.59 J/(mol K), Berman et al. 2007; *S*^o^ = 318 J/(mol K), Holland and Powell 2011). They are estimated ones, based e.g. on the assumption that $S^{\text{o}}_{\text{Eas}} = S^{\text{o}}_{\text{Phl}} - S^{\text{o}}_{\text{MgO}} - S^{\text{o}}_{\text{SiO2}} + S^{\text{o}}_{\text{Al2SiO5}}$ (Berman et al. 2007). Adopting a similar estimation scheme, Circone and Navrotsky (1992) derived *S*^o^ = 317.4 ± 1.2 J/(mol K). This shows that the use of estimation schemes to come up with unknown entropies of phases and widely used in the derivation of internally consistent thermodynamic data sets (e.g., Holland and Powell 2011), may bear serious errors.

For the enthalpy of formation of Eas we obtained $\Delta H^{\text{o}}_{{{\text{f}},{\text{Eas}}}}$ = − 6360.5 kJ/mol from DFT calculations following the procedure outlined in Benisek and Dachs (2018). The anticipated uncertainty of this value, based on the results obtained in that study, compared to reference values, is ca. ± 7 kJ/mol. Our preferred value for $\Delta H^{\text{o}}_{{{\text{f}},{\text{Eas}}}}$ is, however, − 6358.5 ± 1.4 kJ/mol, which results from the correlation between $\Delta H^{\text{o}}_{{{\text{f}},{\text{Eas}}}}$ and *W*_Phl,Eas_ (Eq. 18) established from evaluating the phase-equilibrium data of Berman et al. (2007) on reaction (16) and setting *W*_Phl,Eas_ to the Δcorr-derived value of 25.4 kJ/mol (mid wave number region). This *W*_Phl,Eas_, arising from the mid wave number region of IR spectra, is preferred, because it is in accordance with that resulting from the calorimetric data of Circone and Navrotsky (1992). If the lower value from the high wave number region was chosen instead (10.3 kJ/mol), $\Delta H^{\text{o}}_{{{\text{f}},{\text{Eas}}}}$ would be − 6352.0 ± 1.4 kJ/mol based on Eq. (18). The evaluation of experimental data complementing those of the MASH system will be required to fix final values of $\Delta H^{\text{o}}_{{{\text{f}},{\text{Eas}}}}$ and *W*_Phl,Eas_ unequivocally. Circone and Navrotsky (1992 derived $\Delta H^{\text{o}}_{{{\text{f}},{\text{Eas}}}}$ = − 6358.2 ± 8.8 kJ/mol from an extrapolated heat of solution value for Eas of 281.4 ± 2.6 kJ/mol and corrected by − 6 kJ for the revised enthalpy of sanidine in Robie and Hemingway (1995). Values in the recent data-base literature for $\Delta H^{\text{o}}_{{{\text{f}},{\text{Eas}}}}$ are, on the other hand, more endothermic by 22–35 kJ ($\Delta H^{\text{o}}_{{{\text{f}},{\text{Eas}}}}$ = − 6324.95 kJ/mol, Berman et al. 2007; $\Delta H^{\text{o}}_{{{\text{f}},{\text{Eas}}}}$ = − 6330.48 kJ/mol, Holland and Powell 2011). This difference is of roughly similar magnitude as Δ*H*_dis_ (34.5 kJ/mol), so that, based on the thermodynamic analysis of this study, the data-base values in fact represent the $\Delta H^{\text{o}}_{\text{f}}$ of disordered Eas. The reason for this is simply the use of the by 8% wrong *S*^o^ of Eas in published work intending to extract $\Delta H^{\text{o}}_{{{\text{f}},{\text{Eas}}}}$ from phase equilibrium data obtained on reactions like Eq. (16). As both quantities are linked via Eq. (18), a change in *S*^o^ of Eas will affect its $\Delta H^{\text{o}}_{{{\text{f}},{\text{Eas}}}}$. If we would set arbitrarily $S^{\text{o}}_{\text{Eas}}$ = 318 J/(mol K) and reevaluate these phase-equilibrium data of Berman et al. (2007), we would get $\Delta H^{\text{o}}_{{{\text{f}},{\text{Eas}}}}$ = − 6338 kJ/mol. We note that in the earlier data set issue of Holland and Powell (1998) an estimated $S^{\text{o}}_{\text{Eas}}$ = 306 J/(mol K) was used, more close to the value of this study. With a value of $\Delta H^{\text{o}}_{{{\text{f}},{\text{Eas}}}}$ = − 6348.94 ± 4.70 kJ/mol, the $\Delta H^{\text{o}}_{{{\text{f}},{\text{Eas}}}}$ extracted there is not surprisingly in better agreement with our derived values for $\Delta H^{\text{o}}_{{{\text{f}},{\text{Eas}}}}$. Concerning the reliability of the thermodynamic data of phases with estimated entropies in various data set issues published over time, the above consideration shows that the general belief ‘newer = better’ may be illusory.

### Features of the new activity model for Mg–Al biotite

#### Choice of biotite end-members: does Al order on M1 or on M2 ?

A first step in formulating an activity model is to define appropriate end-members and their site-distributions, which then also defines the ideal activity expressions. As Mg–Al biotites are formed by the Tschermak substitution ${\text{Al}}^{{{\text{oct}}}} {\text{Al}}^{{{\text{tet}}}} {\text{Mg}}^{{{\text{oct}}}} _{{ - 1}} {\text{Si}}^{{{\text{tet}}}} _{{ - 1}}$, mixing of Al and Si on the four tetrahedral (T-) sites is coupled with mixing of Mg and Al on the three octahedral (M-) sites (one M1, two M2-sites, where M1 is slightly larger than M2, e.g., Brigatti and Guggenheim 2002). In published biotite activity models it is generally assumed that T-site mixing is restricted to only two of the four T-sites (T1), in order to maintain Al-avoidance (Holland and Powell 1990, 1998, 2006; Powell and Holland 1999; White et al. 2000, 2007, 2014; B07; Tajčmanová et al. 2009). The site occupancy for end-member Phl is thus straightforward with Mg fully occupying M1 and M2, and Al and Si with equal site-fractions = ½ on the two T1-sites (Table 4). In the Eas end-member, one Al-atom is distributed over the three M-sites and T2 is fully occupied by Al. For a complete disordered arrangement this means that Al occupies the M1 and M2 sites with equal site fractions of *X*_Al_^M1^ = *X*_Al_^M2^ = 1/3. An ordered Eas end-member can be defined in two possible ways: one with Al on M1 (Eas-M1–KAl(Mg_2_)[(OH)_2_Al_2_Si_2_O_10_]) and one with Al on one of the two M2 sites (Eas-M2–KMg(Al,Mg)[(OH)_2_Al_2_Si_2_O_10_]) and it is a priori not clear which one is the better choice. Whereas Eas-M2 was used as end-member in Holland and Powell (1990), all later formulations of the ideal activity of the Eas component appearing in the literature are based on end-member Eas-M1. Structural data on octahedral site-preferences in synthetic biotites seem to confirm this choice. Whereas structural refinements of Fe–Al biotite point to Al^oct^ occupying the two M2 sites (Redhammer et al. 2000; Redhammer and Roth 2002), the IR and Raman spectra of Mg–Al biotite indicate that Al^oct^ orders onto the M1 site (Circone and Navrotsky 1992). Structural data obtained on natural biotites are controversial and do not yield a clear picture on the octahedral site occupancy of Al. Cruciani and Zanazzi (1994) and Brigatti et al. (2000) argue for high-charge cations like Al^3+^ occupying the M2 site. Ventruti et al. (2009), on the other hand, found a preference of Al for the M1 site in a volcanic Fe–Ti bearing phlogopite.

We have performed DFT calculations both on Eas-M1 and Eas-M2 configurations and have found that the CASTEP energy computed for Eas-M1 is lower than most Eas-M2 energies. From the energetical point of view, Eas-M1 should thus be preferred. There is, however, one Eas-M2 configuration that has an equally low energy as Eas-M1. So, also from the CASTEP energies, no clear distinction can be made which of the two Eas end-members is the better choice.

In order to model Mg–Al ordering in biotite, Eas-M1 with Al^oct^ on M1 is, however, the appropriate obvious choice, whereas Eas-M2 with Al and Mg on M2 would require a splitting of the M2 sites in order to constitute an ordered end-member. The relatively large value that we obtained for the disordering enthalpy (ΔH_dis_ = 34.5 ± 3 kJ/mol) may be taken as an indication that Mg–Al ordering in biotite is to be expected as an important crystal-chemical mechanism during cooling, e.g. along a retrograde metamorphic PT-path.

#### *W*_Phl,Eas_ compared to literature data

In this study we determined *W*_Phl,Eas_ of the Phl–Eas (ordered) binary from two independent sources, (a) DFT calculations and (b) line broadening in IR spectra. Both methods indicate positive deviation from ideality characterised by the interaction parameters *W*_Phl,Eas_^DFT^ = 9.1 ± 1.2 kJ/mol and *W*_PhlEas_^Δcorr^ = 10.3 kJ/mol (high wave number region), or *W*_PhlEas_^Δcorr^ = 25.4 kJ/mol (mid wave number region) (Table 7). The latter value is preferred, because it is in accordance with *W*_Phl,Eas_ resulting from the calorimetric data of Circone and Navrotsky (1992) (Fig. 7) and reproduces, in combination with $\Delta H^{\text{o}}_{{{\text{f}},{\text{Eas}}}}$ = − 6358.5 kJ/mol, the experimentally determined Al-saturation level of 1.6 (Al^VI^ = 0.3) of Mg–Al biotite in the assemblage biotite-sillimanite-sanidine-quartz (Berman et al. 2007). If *W*_PhlEas_^Δcorr^ = 10.3 kJ/mol (high wave number region) was chosen instead, $\Delta H^{\text{o}}_{{{\text{f}},{\text{Eas}}}}$ would amount to − 6352.0 ± 1.4 kJ/mol based on Eq. (18). Such a *W*_Phl,Eas_ around 10 kJ/mol, as used in the biotite activity models of Powell and Holland (1999), or Holland and Powell (2006), and adopted in White et al. (2000, 2007, 2014) or Tajčmanová et al. (2009), represents local charge balance.

**Table 7:** Mixing properties of Mg–Al biotite as determined in this study

Join		Parameter source	*W*_H,i,j_^a^		*W*_S,i,j_ calorimetry [J/(mol K)]
			Δcorr (kJ/mol)	DFT (kJ/mol)	
*i *–*j*					
Phl–Eas			**25.4/**10.3^b^	9.1 ± 1.2^c^	**0**
Phl–dEas				**0** ^d^	
Eas–dEas^e^				**0**	

End-members are phlogopite (Phl), ordered eastonite (Eas) and disordered eastonite (dEas), as defined in Table 4. Parameter values used in the activity model are in bold face

^a^For simplicity, the subscript ‘H’ has been omitted in the text, i.e., *W*_H,Phl,Eas_ in the table corresponds to *W*_Phl,Eas_ of text

^b^Derived from Δcorr values given in Table 1 for the mid/high wavenumber regions of 400–600 cm^−1^ and 790–1330 cm^−1^

^c^Minimum value for local charge balance (see text)

^d^See text for further details

^e^The enthalpy change from ordered to disordered Eas amounts to Δ*H*_dis_ = 34.5 ± 3 kJ/mol

As noted above, such a *W*_Phl,Eas_ should be considered as minimum non-ideality along the Phl–Eas join. It represents the structural situation in which defect pairs having only 1st next nearest distances of 3.2 Å to each other. Counting the number of placing defect-atom pairs with 1st, 2nd and 3rd next nearest distances, one finds that there are four different realisations of pairs with 1st next nearest, four of 2nd and eight of 3rd next nearest distances. For the disordered state it is reasonable to expect that all these possibilities are realised. Assuming that the correlation shown in Fig. 6 (Eq. 13), relating distance to *W*_Phl,Eas_^DFT^, applies as a general energetic model for Mg–Al biotites, we compute a mean *W*_Phl,Eas_^DFT^ of 35.1 kJ/mol, a value that is identical to Δ*H*_dis_ = 34.5 ± 3 kJ/mol. This implies to set *W*_Phl,dEas_ = 0, i.e., the line representing ideality in the Phl–dEas binary is a tangent at the Phl-side of the Phl–Eas binary to a Δ*H*_mix_ curve constructed using a *W*_Phl,Eas_ that equals Δ*H*_dis_ (Fig. 7). In other words, a thermodynamic description of the Phl–Eas binary with a *W*_Phl,Eas_ in the order of the magnitude of Δ*H*_dis_ is equivalent to one of the Phl–dEas binary with *W*_Phl,dEas_ = 0 kJ/mol in the Phl-rich part of the system. With increasing Tschermak substitution, Mg–Al ordering becomes successively relevant, which can be simulated using the two Eas end-members, ordered and disordered, in our ternary Mg–Al biotite activity model. Our proposed W_Phl,Eas_ value of 25.4 kJ/mol is considerably larger than 9.1 ± 1.2 kJ/mol—this value corresponds to a strict local charge balance structural situation—and is consistent with the use of MOS ideal activities, as it represents not only 1st next nearest distances between octahedral and tetrahedral defects, but also the contribution of defects with 2nd and 3rd next nearest mutual distances.

#### Activity: composition relationships

**Fig. 8:**
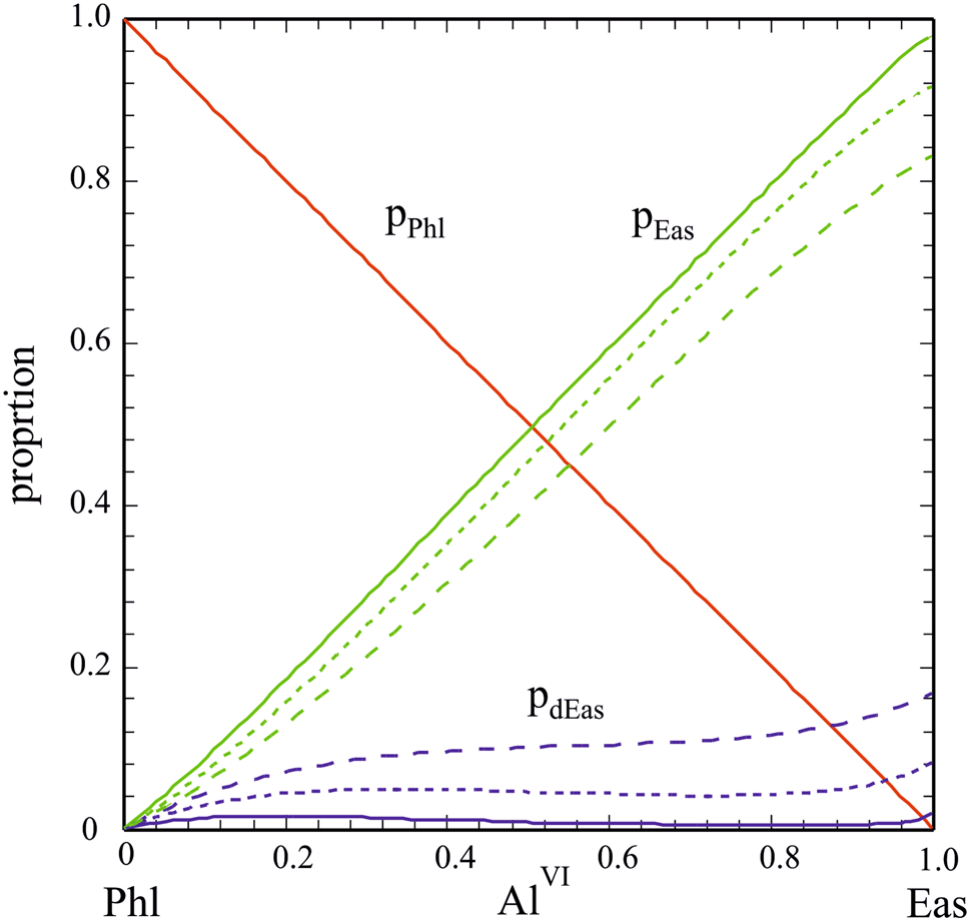
Proportions of the end-members Phl, Eas and dEas as function of the Al^VI^-content in Mg–Al biotite for various temperatures (solid: 400 °C, short-dashed: 700 °C, long-dashed: 1000 °C)

**Fig. 9:**
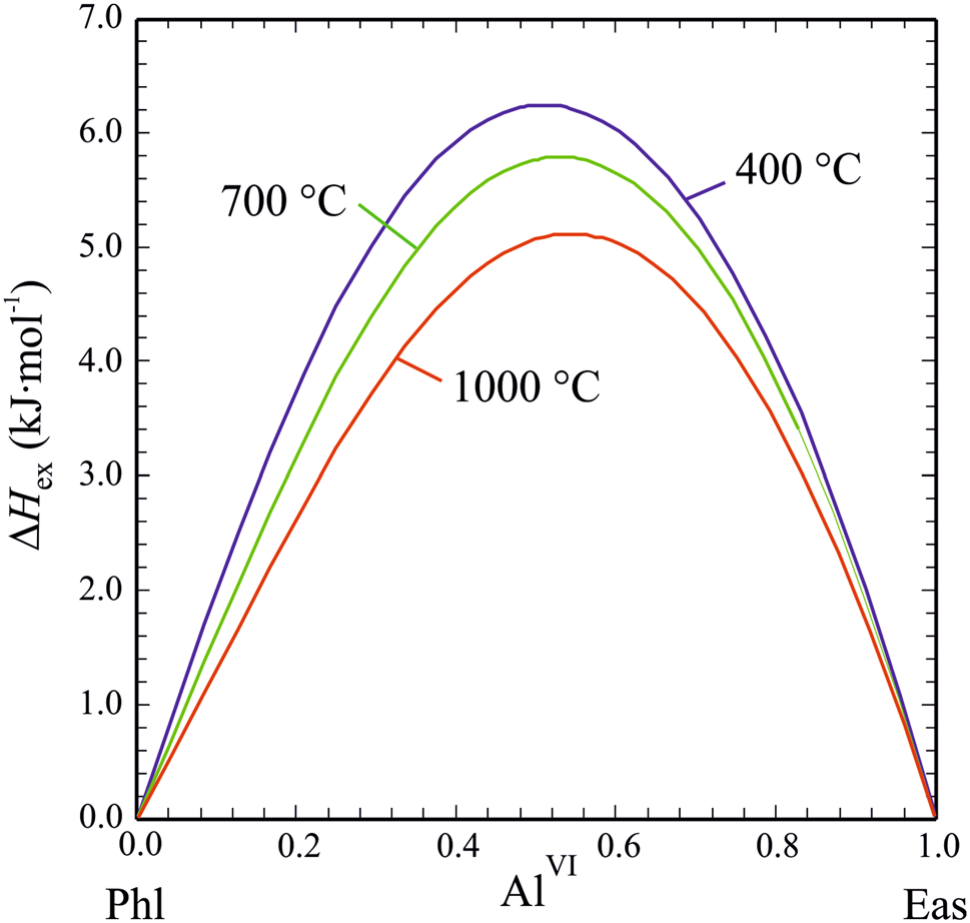
Ternary excess enthalpies of mixing, Δ*H*_ex_ (Eq. 19), as function of the octahedral Al-content, Al^VI^, in Mg–Al biotite for temperatures of 400 °C, 700 °C and 1000 °C

The proportions of the Phl, Eas and dEas end-members are plotted in Fig. 8 for 400 °C, 700 °C and 1000 °C, respectively, as function of Al^VI^. In this temperature range, the proportion of dEas is always < ca. 0.16 with a weak maximum at low *T*’s at Phl-rich compositions. Ternary Δ*H*_ex_, given by the relation (e.g., Ganguly 2008):

19 $$\begin{aligned} \Delta H_{{{\text{ex}}}}^{{{\text{ternary}}}} & = p_{{{\text{Phl}}}} p_{{{\text{dEas}}}} W_{{{\text{Phl}},{\text{dEas}}}} + p_{{{\text{Phl}}}} p_{{{\text{Eas}}}} W_{{{\text{Phl}},{\text{Eas}}}} \\ & \quad + p_{{{\text{Eas}}}} p_{{{\text{dEas}}}} W_{{{\text{Eas}},{\text{dEas}}}} , \\ \end{aligned}$$

is shown in Fig. 9 for 400 °C, 700 °C and 1000 °C, computed using *W*_Phl,Eas_ = 25.4 kJ/mol and *W*_Phl,dEas_. = *W*_Eas,dEas_. = 0 kJ/mol (Table 7). Depending on temperature, Δ*H*_ex_^ternary^ reaches maximal values between ca. 6.3 kJ/mol (at 400 °C) and 5.1 kJ/mol (at 1000 °C), whereby this maximum shifts slightly from Al^VI^ ~ 0.5 towards Al-richer compositions. The predicted Δ*H*_ex_-Al^VI^ behaviour is in good agreement with the solution calorimetric data of Circone and Navrotsky (1992), especially at Al^VI^ < 0.5.

**Fig. 10:**
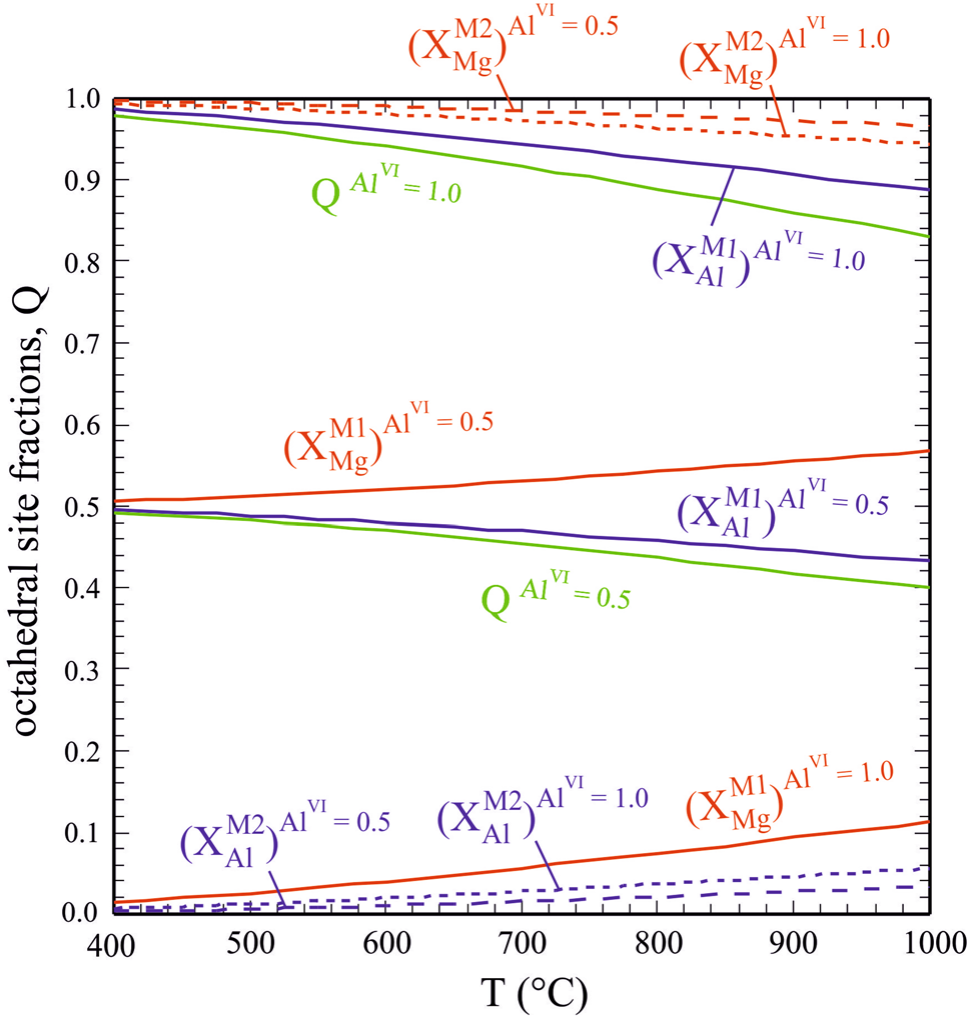
Octahedral site fractions in Mg–Al biotite and order parameter *Q* as function of temperature for constant Al^VI^-contents of 1.0 and 0.5 (that is, *X*_Al_^M1^ + *X*_Al_^M2^ = 1 and 0.5)

In order to illustrate the extent of Mg–Al ordering in biotite, the order parameter Q and site fractions of Mg and Al on the M1 and M2 sites of biotite are shown in Fig. 10 as function of temperature for a bulk Al^VI^-content of 1.0 (Eas composition) and 0.5. For pure Eas, the Mg–Al distribution is close to being completely ordered at 400 °C, i.e. Q, *X*_Al_^M1^ and *X*_Mg_^M2^ are close to unity, whereas the counterparts *X*_Mg_^M1^ and *X*_Al_^M2^ are close to zero (Fig. 10). An increase in temperature leads to a moderate Mg–Al disorder over M1 and M2, characterised by an order parameter *Q* ~ 0.83 at 1000 °C and a *X*_Al_^M1^ that has dropped from ~ 1.0 to ~ 0.9 and vice versa a X_Mg_^M1^ that has raised from ~ 0 to ~ 0.1. To achieve complete disorder, unrealistically high *T*’s would be required. As another example, the change in site fractions as function of temperature is shown in Fig. 10 for an Al^VI^-content of 0.5.

**Fig. 11:**
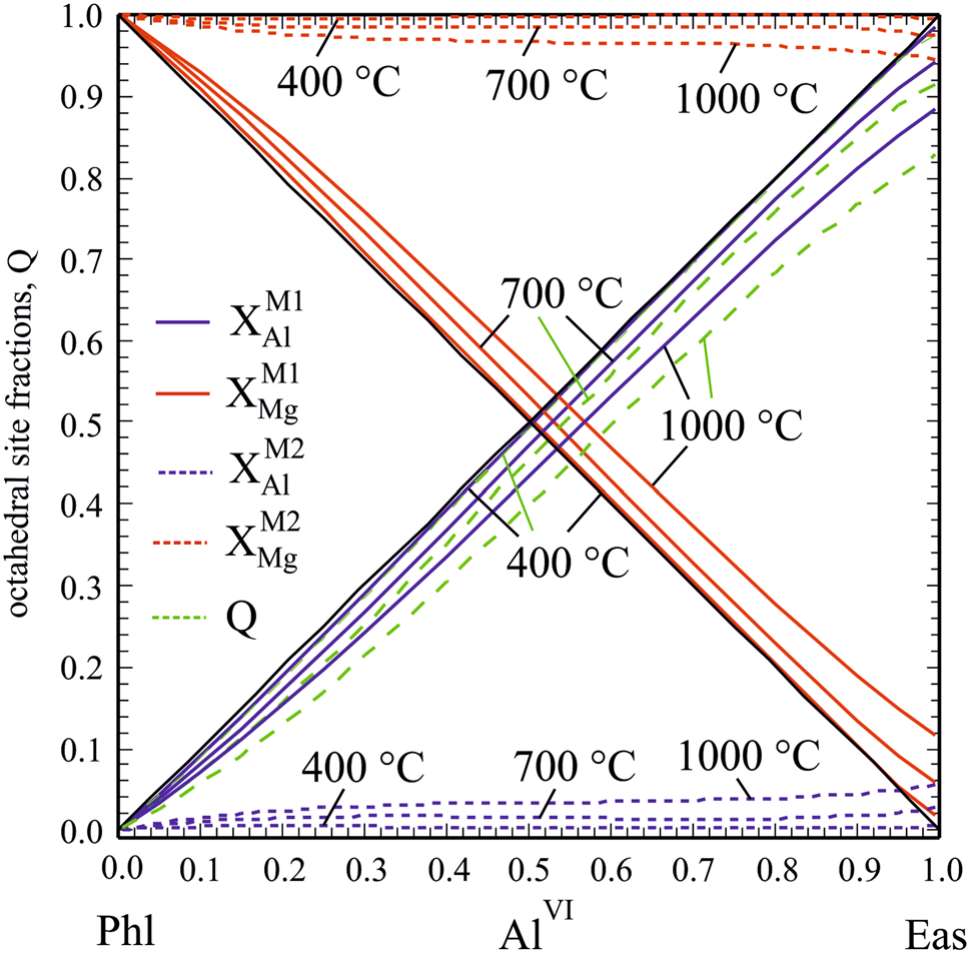
Octahedral site fractions in Mg–Al biotite as function of the Al^VI^-contents for temperatures of 400 °C, 700 °C and 1000 °C

The Mg–Al order/disorder behaviour as function of the Al^VI^-content in Mg–Al biotite is plotted in Fig. 11 for *T*s of 400 °C, 700 °C and 1000 °C. At low *T*s around 400 °C, our activity model converges to these presently used that assume complete Mg–Al order at all temperatures, i.e. all Al^VI^ resides on M1 and M2 is completely filled with Mg so that *Q* = *X*_Al_^M1^ − *X*_Al_^M2^ = Al^VI^. With increasing temperature, disordering of Al over M1 and M2 leads to a drop of *X*_Al_^M1^ and X_Mg_^M2^ (half that of *X*_Al_^M1^, because two M2 sites are involved), as well as of Q, and a corresponding increase in their counterparts X_Mg_^M1^ and *X*_Al_^M2^. An inspection of Fig. 11 shows that this change in site fractions due to an increase in temperature from 400 to 1000 °C is relatively moderate and is around 0.1 for M1 and 0.05 for M2 sites at maximum for the Eas composition (i.e., *X*_Al_^M1,400^ ^°C^ = 0.986, *X*_Al_^M1,1000^ ^°C^ = 0.888, *X*_Al_^M2,400^ ^°C^ = 0.007, *X*_Al_^M2,1000^ ^°C^ = 0.056). With decreasing Al^VI^ content in the Mg–Al biotite, these relative changes in site fractions decrease accordingly.

**Fig. 12:**
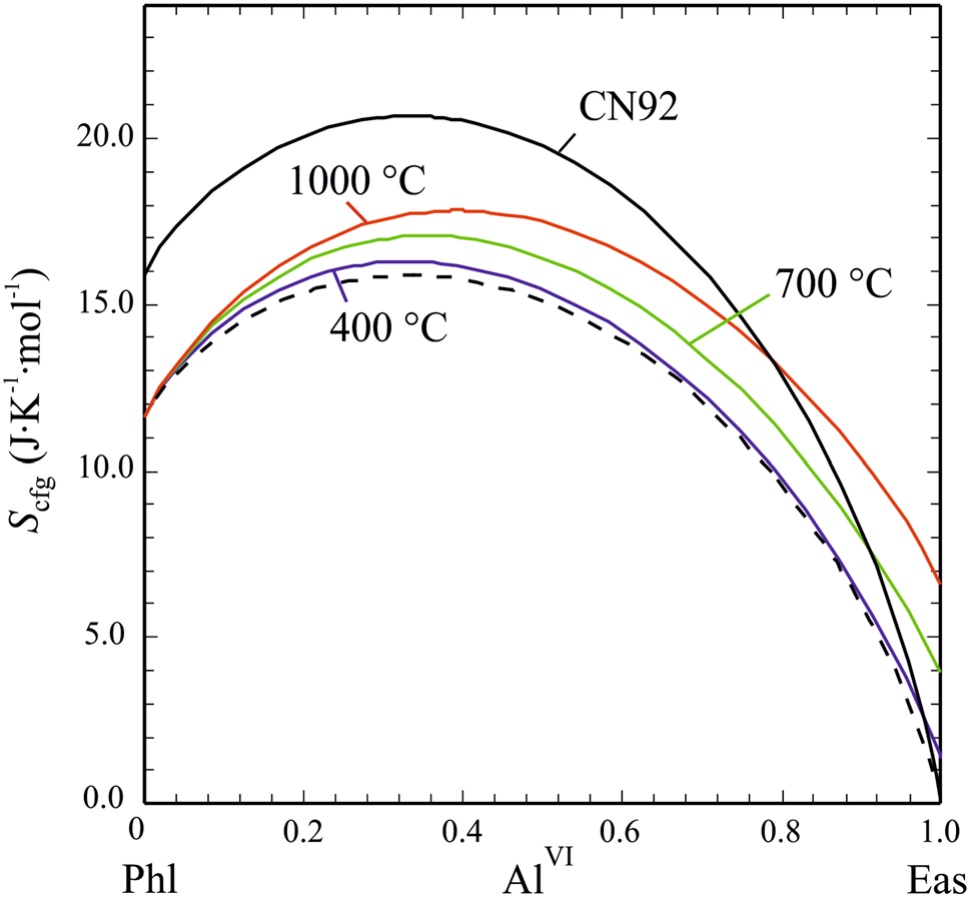
Configurational entropy, *S*_cfg_, as function of the octahedral Al-content, Al^VI^, in Mg–Al biotite for temperatures of 400 °C, 700 °C and 1000 °C. *S*_cfg_ according to Circone and Navrotsky (1992, their Eq. 14—curve labelled ‘CN92’) and as used in present Mg–Al biotite activity models (dashed curve) is also shown

To complete the description of our new activity model for Mg–Al biotite, the configurational entropy, S_cfg_, along the Phl–Eas join, is compared in Fig. 12 to that proposed earlier by Circone and Navrotsky (1992) and that resulting from the biotite activity model of Powell and Holland (1999) and subsequent versions (White et al. 2000, 2007, 2014; Holland and Powell 2006). As disorder increases with temperature, *S*_conf_ gets larger. This temperature dependence vanishes at Phl-rich compositions where the solid-solution is highly Mg–Al disordered.

**Fig. 13:**
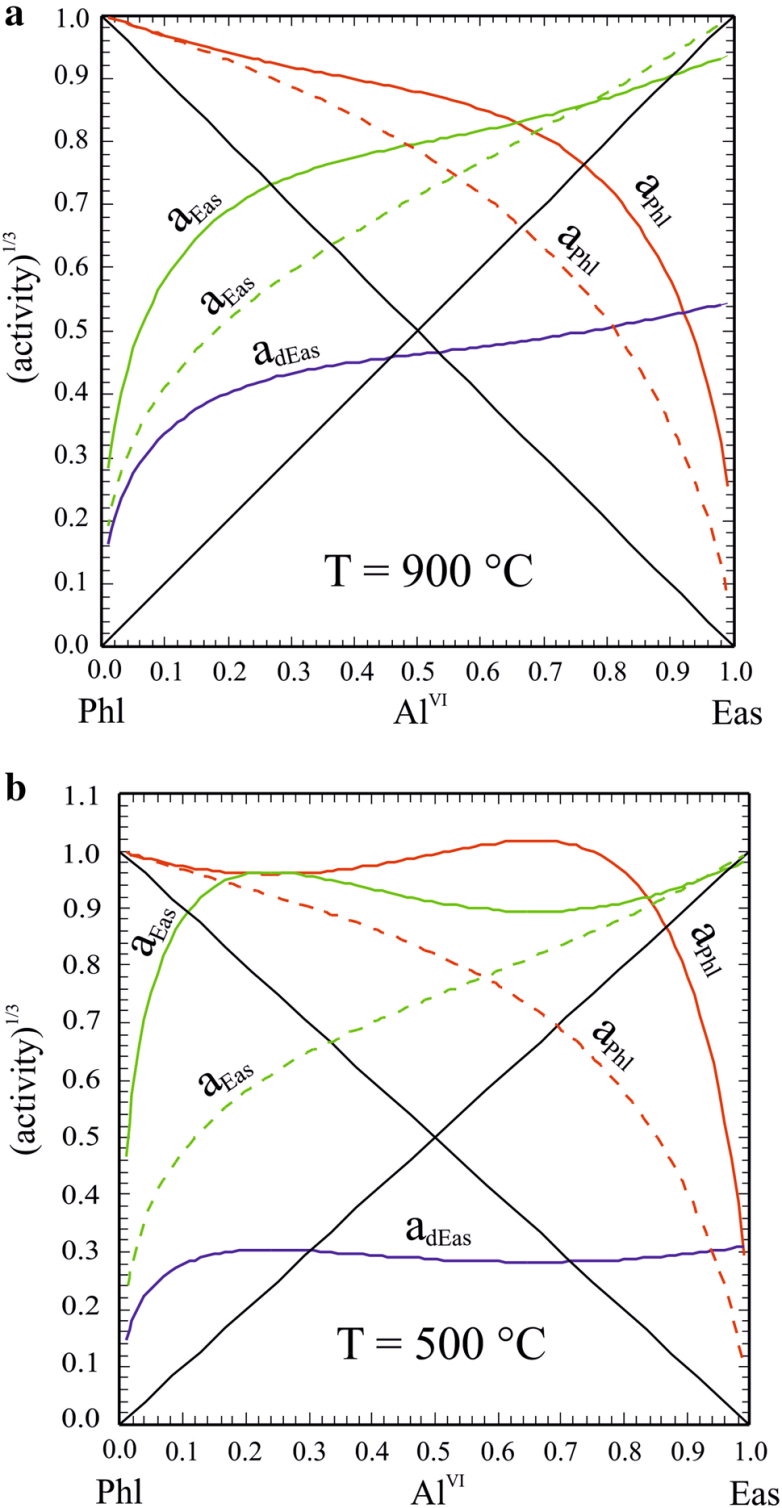
Activities of the Phl, Eas and dEas end-members as function of the Al^VI^ content in Mg–Al biotite for **a** 700 °C and **b** 500 °C, computed with mixing parameters from Table 7 (solid curves), compared to using W_Phl,Eas_ = 10 kJ/mol (dashed curves)

In terms of activity–composition relationships (Fig. 13a, b), Phl and Eas activities are generally larger compared to existing activity models. Below ca. 650 °C, minima develop in the Phl and Eas activity curves (Fig. 13b) meaning that there are two compositions with equal activity, which results in a symmetric miscibility gap with an apex at 650 °C at Al^VI^ = 0.49. At 500 °C, a Phl-rich biotite with 12 mol % Eas component coexists with an Eas-rich one having the same amount of Phl component. Our activity model thus predicts unmixing of a homogenous Mg–Al biotite below ca. 650 °C. This is consistent with all of our synthesis runs, performed at 700 °C, in which no peak splitting or broadening in XRD patterns as indication of the presence of two micas could be observed. This fact may also serve to constrain an upper limit of *W*_Phl,Eas_. Values of this mixing parameter larger than ~ 28 kJ/mol would produce a gap with a crest at temperatures higher than 750 °C and the synthesis runs Phl60Eas40, Phl50Eas50 and Phl40Eas60 would lie well within the miscibility gap. As no signs of unmixing could be found in these experiments, this limits *W*_Phl,Eas_ to ~ 28 kJ/mol.

## Application

### Recalculating the Al-saturation level of Mg–Al biotite in the assemblage (Mg–Al)-biotite-sillimanite-sanidine-quartz

From their experimental brackets, Berman et al. (2007) defined an Al-saturation level of 1.60 ± 0.04 over the *P*–*T* range 650–750 °C and 1.1–3.4 kbar for biotite in the assemblage (Mg–Al)-biotite-sillimanite-sanidine-quartz (+ H_2_O). This corresponds to Al^VI^ = 0.3 ± 0.02 (i.e., (1.6 − 1)/2). Recalculating this experimentally determined Al^VI^ with the above discussed values $\Delta H^{\text{o}}_{{{\text{f}},{\text{Eas}}}}$ = − 6358.5 kJ/mol and *W*_Phl,Eas_ = 25.4 kJ/mol, we recalculate an Al-saturation level of 1.58 at 700 °C (Al^VI^ = 0.29). With increasing temperature, the Al-saturation level decreases in accordance with experimental evidence to values of 1.31 and 1.23 at 800 °C and 900 °C, respectively. Repeating the same calculation with all thermodynamic data and functions according to Holland and Powell (2011), with the similar biotite activity model as described, e.g. in White et al. (2000, 2007, 2014), we obtain an Al saturation level of 1.80 (Al^VI^ = 0.4) at 700 °C.

### Mg–Al ordering during cooling of a volcanic phlogopite

Ventruti et al. (2009) investigated the cation partitioning in a natural Fe- and Ti-bearing volcanic phlogopite by in-situ neutron powder diffraction and FTIR spectroscopy. This phlogopite contained 0.17 ± 0.01 formula units Al^VI^, and from a Rietfeld refinement of their neutron data Ventruti et al. (2009) derived octahedral site occupancies of *X*_Al_^M1^ = 0.17 ± 0.03 and *X*_Al_^M2^ = 0.00 ± 0.02. Applying our activity model to this case yields the M1 site fractions *X*_Al_^M1,400^ ^°C^ = 0.15, *X*_Al_^M1,1000^ ^°C^ = 0.12 and the M2 site fractions *X*_Al_^M2,400^ ^°C^ = 0.01, *X*_Al_^M2,1000^ ^°C^ = 0.03. The values at 400 °C agree with those of Ventruti et al. (2009) within 2*σ*-error and can be interpreted on the basis of our activity model to have developed from the high-*T* site fractions due to Mg–Al ordering on cooling.

### Eas + Qz and Phl + Qz stability

The dehydration of Eas in the presence of Qz is given by the following reaction:

20 $$2\;{\text{Eastonite}} + 4\;{\text{quartz}} = {\text{enstatite}} + 2\;{\text{sanidine}} + 2\;{\text{spinel}} + 2\;{\text{H}}_{ 2} {\text{O}} .$$

**Fig. 14:**
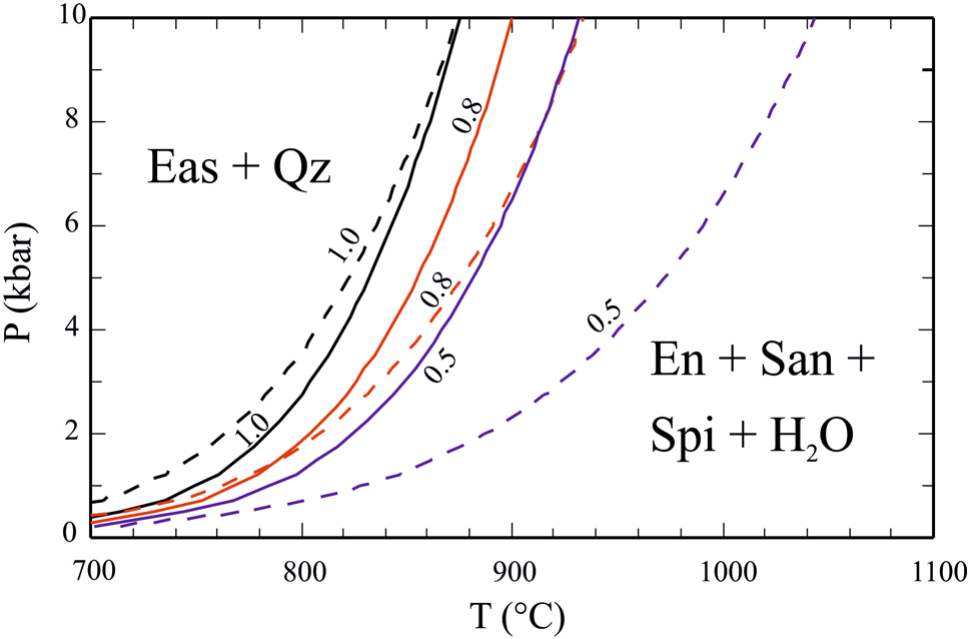
*P*–*T* positions of reaction (27), limiting Eas + Qz stability, for Al^VI^ contents in Mg–Al biotite of 1.0, 0.8 and 0.5 (numbers on curves), calculated with mixing parameters from Table 7 and eastonite’s standard state data of this study (Table 8, solid curves), compared to calculations using W_Phl,Eas_ = 10 kJ/mol and the Holland and Powell (2011) data set (dashed curves)

Its $\Delta S^{\text{o}}_{\text{R}}$ and $\Delta H^{\text{o}}_{\text{R}}$ are 348 J/(mol K) and 250.2 kJ/mol, respectively, based on the revised *S*_o_ and $\Delta H^{\text{o}}_{\text{f}}$ values of Eas(ordered) of this study, as summarised in Table 8, and taking all other thermodynamic data from Holland and Powell (2011). If also the data for end-member Eas were used from this data base, $\Delta S^{\text{o}}_{\text{R}}$ and $\Delta H^{\text{o}}_{\text{R}}$ would amount to 301 J/(mol K) and 194.0 kJ/mol, respectively. The *P*–*T* position of reaction (20) is shown in Fig. 14 for activities of the Eas component at Al^VI^ contents of 1.0, 0.8 and 0.5, respectively, using both sets of eastonite’s *S*^o^ and $\Delta H^{\text{o}}_{\text{f}}$ values and computing Eas activity with either the model of this study with biotite mixing properties from Table 7 (case-1), or setting *W*_Phl,Eas_ = 10 kJ/mol (e.g., Powell and Holland 1999; Holland and Powell 2006—case-2). Due to the larger $\Delta S^{\text{o}}_{\text{R}}$, case-1 curves have steeper slopes. As a response to lowering the Al^VI^–content in eastonite by dilution with Phl-component, the stability field of such a Mg–Al biotite is less expanded to higher temperatures, because Eas activity is generally larger here compared to case-2 (Fig. 13).

**Table 8:** Standard state (1 bar, 298.15 K) thermodynamic properties of the biotite end-members phlogopite (Phl) and ordered eastonite (Eas) as derived in this study

	$\Delta H^{\text{o}}_{\text{f}}$ (kJ/mol)	*S*^o^ [J/(mol K)]	*V*^o^ [J/(mol bar)]	*k*_0_	*k*_1_	*k*_2_ × 10^-7^	*k*_3_ × 10^-9^
Phl	− 6209.83 ± 1.10	330.9 ± 2.2^a^	14.96	667.37	− 3914.50	− 1.52396	2.17269
Eas	− 6358.50 ± 1.40^b^	294.5 ± 3.0	14.65	656.91	− 3622.01	− 1.70983	2.31802

*C*_p_ = *k*_0_ + *k*_1_·*T*^−0.5^ + *k*_2_·*T*^−2^ + *k*_3_·*T*^−3^ [J/(mol K)]

^a^The calorimetric entropy of Phl at 298.15 K is 319.4 ± 2.2 J/mol K to which *S*_cfg_ = 11.5 J/mol K is added

^b^Based on *W*_PhlEas_^Δcorr^ = 25.4 kJ/mol (IR spectra, mid wave number region) and the correlation between $\Delta H^{\text{o}}_{{{\text{f}},{\text{Eas}}}}$ and *W*_Phl,Eas_ (Eq. 18). *W*_PhlEas_^Δcorr^ = 10.3 kJ/mol from the high wave number region would yield $\Delta H^{\text{o}}_{{{\text{f}},{\text{Eas}}}}$ = − 6352.0 kJ/mol

**Fig. 15:**
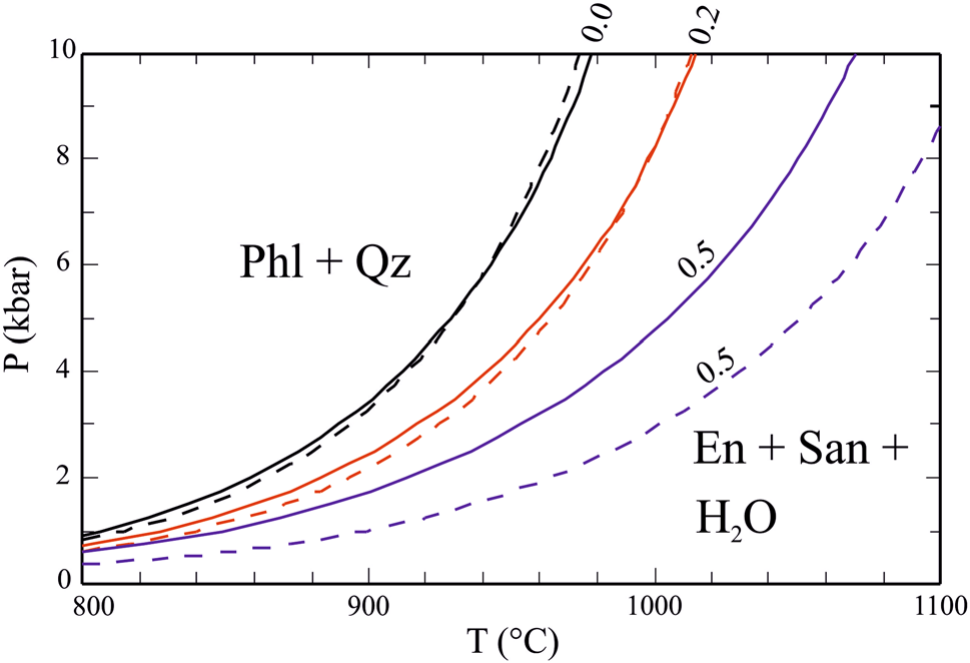
*P*–*T* positions of reaction (5), limiting Phl + Qz stability, for Al^VI^ contents in Mg–Al biotite of 0.0, 0.2 and 0.5 (numbers on curves), calculated with mixing parameters from Table 7 and phlogopite’s standard state data of this study (Table 8, solid curves), compared to calculations using *W*_Phl,Eas_ = 10 kJ/mol and the Holland and Powell (2011) data set (dashed curves)

For phlogopite + quartz stability, thermally limited by reaction (7), we find an analogous situation, as shown in Fig. 15. Here, break-down reaction (7) was computed for pure Phl and for Tschermak-substituted phlogopites with Al^VI^ = 0.2 and 0.5. As a consequence of the new activity model and phlogopite’s revised *S*^o^ and $\Delta H^{\text{o}}_{\text{f}}$ of this study (Table 8), the stability field of Mg–Al biotite + quartz is less expanded to higher *T*s compared to its shape using the data base values of, e.g. Holland and Powell (2011) and *W*_Phl,Eas_ = 10 kJ/mol in the phase-equilibrium calculations.

## Conclusions

In this contribution, we have applied an integrated approach to construct a new activity model for biotite in the KMASH system. This approach combines all information available for this solid solution from branches like calorimetry, line-broadening in IR spectra, DFT calculations and evaluation of phase-equilibrium data. The resulting model (with mixing parameters given in Table 7) thus has a sound physical basis.

The application of DFT allowed us to derive a value for the disordering enthalpy associated with the disordering of Mg and Al on the M sites of eastonite (Δ*H*_dis_ = 34.5 ± 3 kJ/mol). This value is reliable, as from our experience so far, DFT computations yield reasonable results in agreement with experimental evidence, especially in cases of phase-transition enthalpies like, e.g. α-β quartz (Dachs et al. 2018). Additionally, DFT calculations are a promising new tool in geosciences for deriving standard state thermodynamic properties of mineral end-members (Benisek and Dachs 2018). By applying the single-defect method (Sluiter and Kawazoe 2002), we constrained a minimum value of *W*_Phl,Eas_ = 9.1 kJ/mol, corresponding to strict local charge balance, for the Phl–Eas join and it will be demonstrated in a forthcoming paper that mixing properties of solid solutions can be surprisingly well predicted applying this technique (Benisek and Dachs, in prep.). Based on the extent of line-broadening in IR spectra, *W*_Phl,Eas_ is of comparable magnitude considering line-broadening in the high wave number region (10.3 kJ/mol), or larger amounting to 25.4 kJ/mol (mid wave number region). The latter value is preferred at the moment, because it is in accordance with Δ*H*_ex_ from the solution-calorimetric data of Circone and Navrotsky (1992), Applying it to KMASH phase-equilibrium data of Berman et al. (2007), leads to a $\Delta H^{\text{o}}_{{{\text{f}},{\text{Eas}}}}$ = − 6358.5 ± 1.4 kJ/mol, 2 kJ different to the DFT-derived $\Delta H^{\text{o}}_{{{\text{f}},{\text{Eas}}}}$ (Table 8). The evaluation of phase-equilibrium data complementing those available for the KMASH system will be required to fix values of $\Delta H^{\text{o}}_{{{\text{f}},{\text{Eas}}}}$ and *W*_Phl,Eas_ unequivocally.

Low-temperature heat capacity measurements on synthetic pure Phl enabled us to provide a revised value for *S*^o^ of this end-member (330.9 ± 2.2 J/(mol K) including *S*_cfg_ = 11.5 J/(mol K)). Evaluating phase-equilibrium data on Phl + Qz stability, the 1% larger Phl entropy causes a $\Delta H^{\text{o}}_{{{\text{f}},{\text{Phl}}}}$ that is larger by 7–8 kJ/mol ($\Delta H^{\text{o}}_{{{\text{f}},{\text{Phl}}}}$ = − 6209.83 ± 1.1 kJ/mol) than tabulated values.

Calorimetric entropies at 298.15 K change linearly with composition along the Phl–Eas join, there are thus no excess entropies of mixing in this binary. The linear extrapolation of these calorimetric data yields the standard entropy of *S*^o^ = 294.5 ± 3.0 J/(mol K) for Eas in excellent agreement with the DFT-derived *S*^o^, but ~8% smaller than estimated values as appearing in thermodynamic data bases like Holland and Powell (2011).

In a subsequent contribution, we extend our KMASH biotite activity model to include Fe^2+/3+^ and Ti in order to provide a physically based model for general applicability in petrology.

## Electronic supplementary material

Below is the link to the electronic supplementary material.

Supplementary material 1 (XLS 280 kb)

Supplementary material 2 (DOCX 6063 kb)
