# Supplementary material for: A new activity model for Mg–Al biotites determined through an integrated approach
Source: Contrib Mineral Petrol. 2019 Aug 23;174(9):76. doi: 10.1007/s00410-019-1606-2 (PMC6707958; doi:10.1007/s00410-019-1606-2)
Supplement: Supplementary file 2 — Supplementary material 2 (DOCX 6063 kb) [file 410_2019_1606_MOESM2_ESM.docx]

**Supplementary material** to the study:

“A new activity model for Mg-Al biotites determined through an integrated approach” published in *Contributions to Mineralogy and Petrology* by Edgar Dachs and Artur Benisek, University of Salzburg, Austria.

**

**

**Fig. S1.** SEM image of synthetic phlogopite.


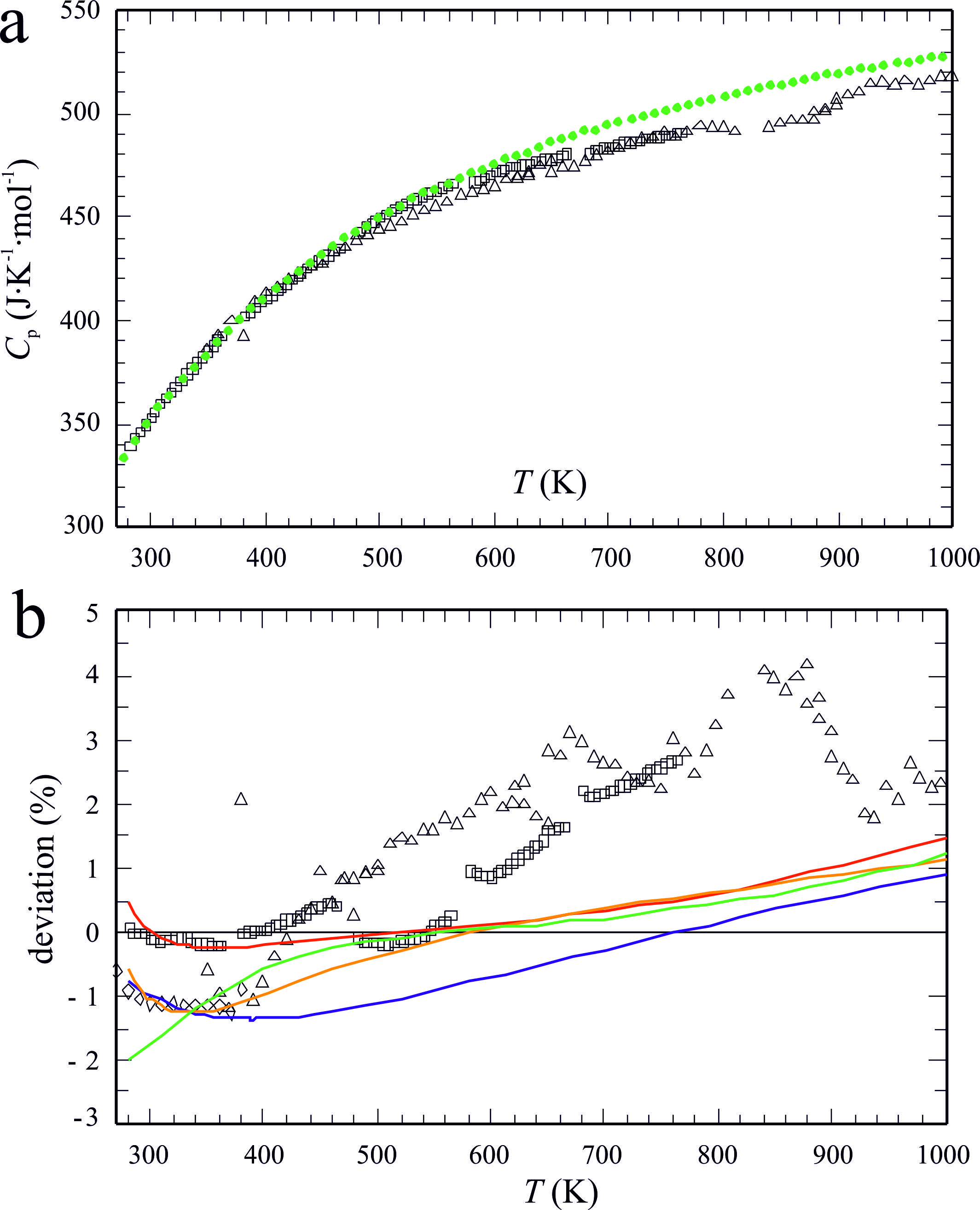


**Fig. S2.** **(a)** DSC-measured (open squares) and DFT-computed (green dots) molar heat capacities of phlogopite in the temperature range 280 – 1000 K from this study (sample Phl100), compared to the DSC data of Robie and Hemingway (1984) (open triangles). **(b)** Deviation of the superambient C_p_’s for phlogopite computed with the polynomial of eq. (5) from C_p_ calculated from published polynomials, i.e. 100(C_p_^eq.(3)^ – C_p_^lit.^)/C_p_^lit.^. Red: Robie and Hemingway (1984, their eq. 2); green: Circone and Navrotsky (1992, their Tab. 1); blue: Berman et al. (2007, their Tab. 5); brown: Holland and Powell (2011, estimated). Diamonds represent the deviation from the adiabatic calorimetric data of Robie and Hemingway (1984).


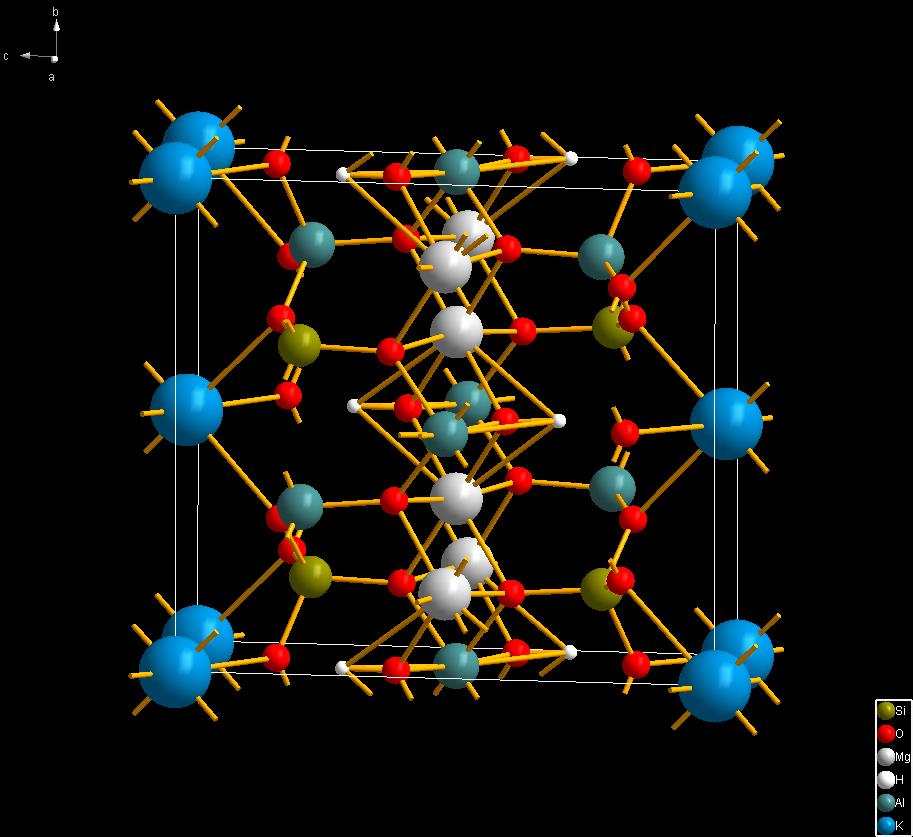

**Fig. S3:** Structure of fully ordered eastonite (Al on M1 and Mg on M2 in the octahedral sheet, Al on T1 and Si on T2 in the tetrahedral sheet).


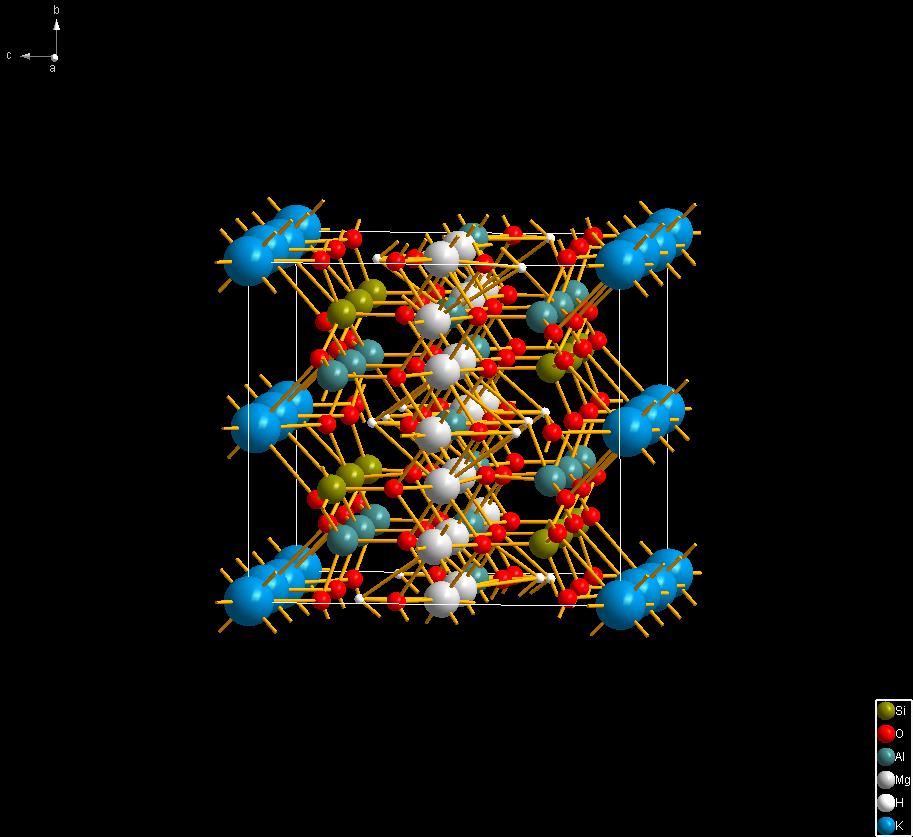


**Fig. S4:** Structure of fully disordered eastonite with Al occupying 1/3 of octahedral M sites. In some rows, 4 octahedral atoms can be seen. Since the first atom is identical with the last, these rows consist of only 3 atoms.


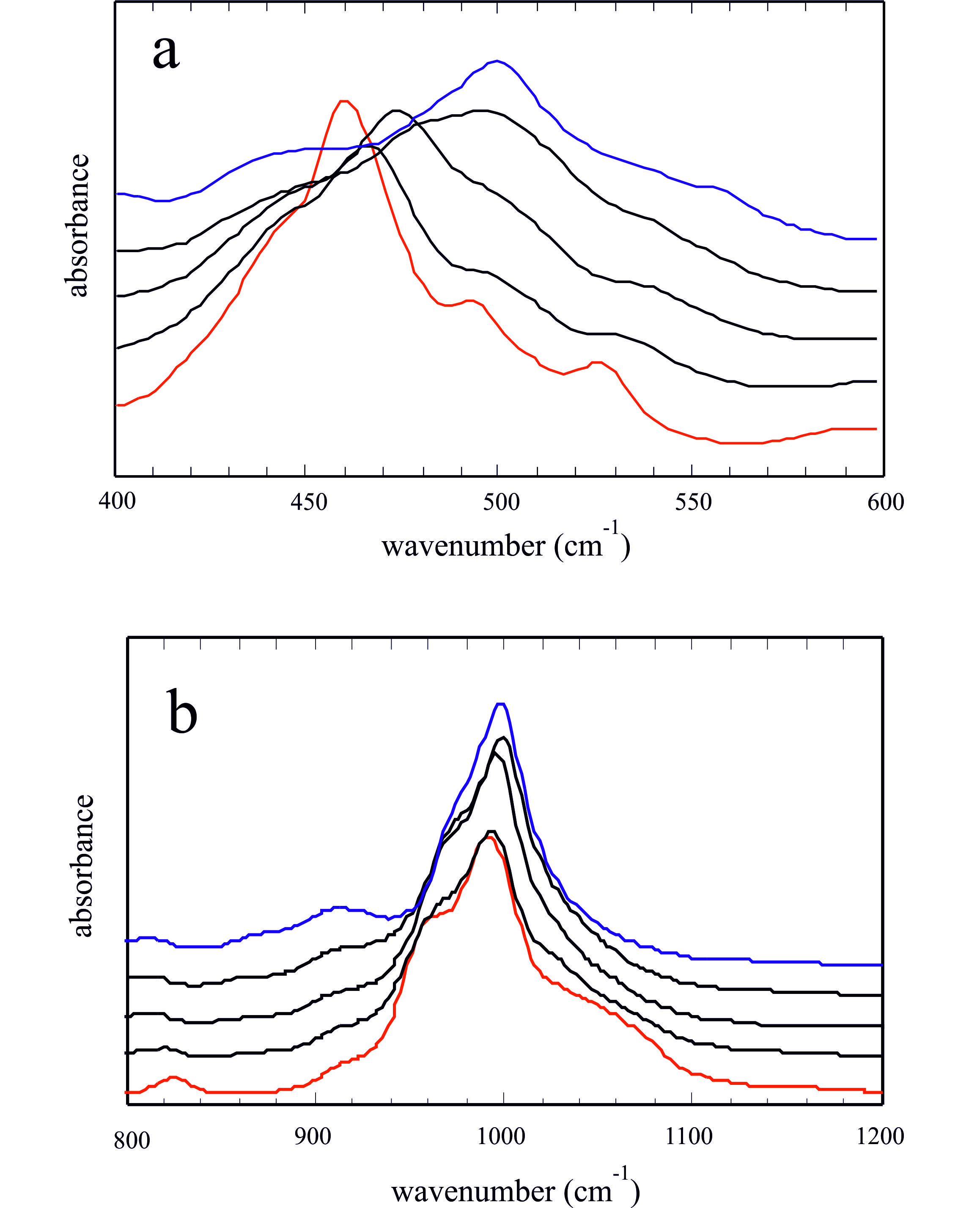


**Fig. S5.** IR spectra of Phl100 (red), Phl80Eas20, Phl60Eas40, Phl40Eas60 and Eas100 (blue) in the wave number region **a)** 400 – 600 cm^-1^ and **b)** 800 – 1200 cm^-1^ (see Tab. 1 for compositions). Autocorrelation analysis to these peaks yields the Δcorr values given in Tab. 1.

**Table S1.** Separate Excel file with the calorimetric data.

**Table S2.** Selected published experimental brackets obtained on the reaction 2 Phl + 6 Qtz = 3 En + 2 San + 2 H_2_O, used to extract the enthalpy of formation of Phl as discussed in the text.

| **Run number** | **P [kbar]** | **T [°C]** | **X_H2O_** | **a_H2O_** | **Stable assemblage** | **Reference** |
| --- | --- | --- | --- | --- | --- | --- |
|  |  |  |  |  |  |  |
| 20 / 22 | 0.46 / 0.49 | 769 / 755 | 1 / 1 |  | En + Sa / Phl + Qtz | Berman et al. (2007) |
| 18 / 19 | 1.74 / 1.74 | 768 / 760 | 0.51 / 0.51^1)^ |  | En + Sa / Phl + Qtz |  |
| PQ3 / PQ1 | 2 / 2 | 780 / 765 | 0.49 / 0.50^1)^ |  | En + Sa / Phl + Qtz |  |
|  |  |  |  |  |  |  |
| PQ-43 / PQ-42 | 12 / 12 | 875 / 875 | 0.642 / 0.655^2)^ | 0.45 / 0.45 | En + Sa / Phl + Qtz | Aranovich and Newton (1998) |
| PQ-16 / PQ-17 | 10 / 10 | 850 / 850 | 0.613 / 0.633^2)^ | 0.42 / 0.42 | En + Sa / Phl + Qtz |  |
| PQ-26 / PQ-27 | 10 / 10 | 800 / 800 | 0.485 / 0.522^2)^ | 0.28 / 0.28 | En + Sa / Phl + Qtz |  |
| PQ-51 / PQ-52 | 10 / 10 | 750 / 750 | 0.428 / 0.455^2)^ | 0.21 / 0.21 | En + Sa / Phl + Qtz |  |
| PQ-22 / PQ-23 | 7 / 7 | 850 / 850 | 0.641 / 0.663^2)^ | 0.51 / 0.51 | En + Sa / Phl + Qtz |  |
| PQ-28 / PQ-29 | 7 / 7 | 800 / 800 | 0.562 / 0.582^2)^ | 0.39 / 0.39 | En + Sa / Phl + Qtz |  |
| PQ-47 / PQ-53 | 7 / 7 | 750 / 750 | 0.412 / 0.484^2)^ | 0.26 / 0.26 | En + Sa / Phl + Qtz |  |
| PQ-37 / PQ-21 | 2 / 2 | 800 / 800 | 0.661 / 0.698^2)^ | 0.70 / 0.70 | En + Sa / Phl + Qtz |  |
|  |  |  |  |  |  |  |
| K-22 / K-37 | 0.510 / 0.611 | 800 / 800 | 1 / 1 |  | En + Sa / Phl + Qtz | Clemens (1995) |
|  |  |  |  |  |  |  |
|  | 5 / 5 | 800 / 780 | 0.35 / 0.35^1)^ |  | En + Sa / Phl + Qtz | Bohlen et al. (1983) |

^1)^ H_2_O – CO_2_ fluid

^2)^ H_2_O – KCl solution

**Derivation of text eq. (10):**

The Mg-Al biotite site fractions as function of the order parameter Q and Al^VI^ are given by:

X_Mg_^M1^ = (1/3)(3 - Al^VI^ - 2Q) (S1a),

X_Al_^M1^ = (1/3)(Al^VI^ + 2Q) (S1b),

X_Mg_^M2^ = (1/3)(3 - Al^VI^ + Q) (S1c),

X_Al_^M2^ = (1/3)(Al^VI^ - Q) (S1d),

X_Al_^T1^ = (1/2)(1 + Al^VI^) (S1e),

X_Al_^M1^ = (1/2)(1 - Al^VI^) (S1f).

The ideal mixing-on-sites (MOS) activities (e.g., Holland and Powell 1998) are:

a^id^_Phl_ = 4X_Mg_^M1^(X_Mg_^M2^)^2^X_Al_^T1^X_Si_^T1^ (S2a),

a^id^_Eas_ = X_Al_^M1^(X_Mg_^M2^)^2^(X_Al_^T1^)^2^ (S2b).

a^id^_dEas_ = (27/4)(X_Mg_^M1^)^(2/3)^(X_Al_^M1^)^(1/3)^(X_Mg_^M2^)^(4/3)^(X_Al_^M2^)^(2/3)^(X_Al_^T1^)^2^ (S2c),

In order to describe thermodynamic non-ideality, we apply the symmetric formalism of Powell and Holland (1993). This gives the following expressions for the activity coefficients γ of Phl, Eas and dEas:

RTlnγ_Phl_ = p_dEas_(1-p_Phl_)W_Phl,dEas_ + p_Eas_(1-p_Phl_)W_Phl,Eas_ - p_dEas_p_Eas_W_Eas,dEas_ (S3a)

RTlnγ_Eas_ = p_Phl_(1-p_Eas_)W_Phl,Eas_ + p_dEas_(1-p_Eas_)W_Eas,dEas_ - p_Phl_p_dEas_W_Phl,dEas_ (S3b)

RTlnγ_dEas_ = p_Phl_(1-p_dEas_)W_Phl,dEas_ + p_Eas_(1-p_dEas_)W_Eas,dEas_ - p_Phl_p_Eas_W_Phl,Eas_ (S3c)

W_Phl,dEas_, W_Phl,dEas_ and W_Eas,dEas_ in these expressions are binary macroscopic symmetric interaction parameters (e.g. Ganguly 2008) and p_Phl_, p_Eas_ and p_dEas_ are proportions (or equivalently mole fractions) of the end-members Phl, Eas and dEas, respectively. These are linked to Q and Al^VI^ via the relations:

p_Phl_ = 1 - Al^VI^ (S4a)

p_Eas_ = Q (S4b)

p_dEas_ = Al^VI^ – Q (S4c).

For the internal reaction (8) equilibrium is given by the relation:

ΔG_dis_ = 0 = ΔH_dis_ – TΔS_dis_ + RTln(a^id^_dEas_/a^id^_Eas_) + RTlnγ_dEas_ - RTlnγ_Eas_ (S5),

where ΔH_dis_ and ΔS_dis_ are the enthalpy and entropy of disordering according to eq. (8), respectively, and ΔS_dis_ is given by the difference of configurational entropies (S^conf^) between disordered and ordered eastonite end-members:

ΔS_dis_ = S^conf^_dEas_ - S^conf^_Eas_ = S^conf^_dEas_ (13).

As S^conf^_Eas_ is zero, ΔS_dis_ equals S^conf^_dEas_, which calculates as S^conf^_dEas_ = -3R[(2/3)ln(2/3) + (1/3)ln(1/3)] = Rln(27/4) = 15.88 J/(mol·K). This leads to the following expression for ΔG_dis_ (eq. (10) of text):

$$\Delta G_{dis}= 0=\Delta H_{dis}+2QW_{Eas,dEas}-W_{Phl,Eas}+W_{Phl,dEas}+{Al}^{VI}\left( W_{Phl,Eas}-W_{Phl,dEas}-W_{Eas,dEas} \right)+$$

$RTln\left[ \frac{\left( 3-{Al}^{VI}-2Q \right)\left( {Al}^{VI}-Q \right)}{\left( 3-{Al}^{VI}+Q \right)\left( {Al}^{VI}+2Q \right)} \right]^{\frac{2}{3}}$ (10)
